# Supplementary material for: Patient-initiated second medical consultations—patient characteristics and motivating factors, impact on care and satisfaction: a systematic review
Source: BMJ Open. 2021 Sep 24;11(9):e044033. doi: 10.1136/bmjopen-2020-044033 (PMC8475134; doi:10.1136/bmjopen-2020-044033)
Supplement: Supplementary data [file bmjopen-2020-044033supp001.pdf]

# Appendix 1: Study characteristics

| Study | Medical specialty | Location | Study design | No. of participants | Characteristics | Motivation | Satisfaction | Diagnosis | Treatment | Prognosis |
|---|---|---|---|---|---|---|---|---|---|---|
| Annandale, 1998[24] | Gynaecology, respiratory, cardiovascular, other | Scotland | Cross-sectional | 307 | | X | | | | |
| Benson, 2001[25] | Ophthalmology | United States | Cross-sectional | 100 | X | X | | | X | |
| Cecon. 2019[26] | Breast cancer | Germany | Cross-sectional | 419 | X | X | | X | X | |
| Clauson, 2002[27] | Breast cancer | United States | Cross-sectional | 231 | X | | | | X | |
| Fuchs, 2017 [28] | Cancer | Germany | Cross-sectional | 36 | X | X | X | | X | |
| Gologorsky, 2013[29] | Ophthalmology | United States | Cross-sectional | 174 | X | X | | | | |

| Study | Medical specialty | Location | Study design | No. of participants | Characteristics | Motivation | Satisfaction | Diagnosis | Treatment | Prognosis |
|---|---|---|---|---|---|---|---|---|---|---|
| Groß, 2017[30] | Breast cancer | Germany | Cross-sectional | 2846 | X | X | | | | |
| Katz, 2017[31] | Breast cancer | United States | Cross-sectional | 304 | X | | | | | |
| Kurian, 2017[32] | Breast cancer | United States | Cross-sectional | 168 | X | | | | | |
| Mellink, 2003[33] | Cancer | Netherlands | Cross-sectional | 212 | X | X | | | | |
| Mellink, 2006[34] | Cancer | Netherlands | Cohort | 403 | X | | | X | X | X |
| Meyer, 2015[35] | Orthopaedics, oncology, haematology, other | United States | Cross-sectional | 6791 | | X | X | X | X | |
| Mordechai, 2015[36] | Haematological cancer | Israel | Cross-sectional | 37 | X | | X | | X | |

| Study | Medical specialty | Location | Study design | No. of participants | Characteristics | Motivation | Satisfaction | Diagnosis | Treatment | Prognosis |
|---|---|---|---|---|---|---|---|---|---|---|
| Morrow, 2009[37] | Breast cancer | United States | Cross-sectional | 378 | X | | | | X | |
| Mustafa, 2002[38] | Fatigue, abdominal pain, chest pain, other | Netherlands | Cross-sectional | 201 | X | X | | X | X | |
| Okamoto, 2013[39] | Cancer, neurology, orthopaedics, other | Japan | Cross-sectional | 149 | X | X | X | X | X | |
| Philip, 2010[40] | Cancer | Australia | Cross-sectional | 17/65* | X | X | X | | | |
| Radhakrishnan, 2017[41] | Prostate cancer | United States | Cross-sectional | 950 | X | X | | | | |
| Ramsey, 2011[42] | Prostate cancer | United States | Cohort | 143/25* | X | | | | X | |
| Sato, 1999[43] | Obstetrics, gynaecology, | Japan | Cross-sectional | 420 | X | | | | | |

| Study | Medical specialty | Location | Study design | No. of participants | Characteristics | Motivation | Satisfaction | Diagnosis | Treatment | Prognosis |
|---|---|---|---|---|---|---|---|---|---|---|
| | gastroenterology, other | | | | | | | | | |
| Schook, 2014[44] | Lung cancer | Netherlands | Cross-sectional | 184 | X | | | X | X | |
| Shmueli, 2016[12] | Orthopaedics, ophthalmology, dermatology, other | Israel | Cross-sectional | 208,366 | X | | | | | |
| Shmueli, 2017[45] | Orthopaedics, ophthalmology, dermatology, other | Israel | Cross-sectional | 344 | | X | X | X | X | |
| Shmueli. 2019 [46] | Orthopaedics, ophthalmology, | Israel | Cross-sectional | 143,371 | X | | | | | |

| **Study** | **Medical specialty** | **Location** | **Study design** | **No. of participants** | **Characteristics** | **Motivation** | **Satisfaction** | **Diagnosis** | **Treatment** | **Prognosis** |
|---|---|---|---|---|---|---|---|---|---|---|
| | dermatology, other | | | | | | | | | |
| Shmueli. 2019a[47] | Orthopaedics, ophthalmology, dermatology, other | Israel | Cross-sectional | 339 | X | X | X | X | X | |
| Sutherland. 1989 [48] | Gastroenterology | Canada | Cross-sectional | 246 | X | X | | | | |
| Sutherland. 1994 [49] | Gastroenterology | Canada | Cross-sectional | 341 | X | | | | | |
| Tam, 2005[50] | Gynaecologic cancer | Hong Kong | Cross-sectional | 80 | X | X | | | | |
| Tan, 2014[51] | Gynaecology | United States | Cross-sectional | 205 | X | | | X | X | |

| Study | Medical specialty | Location | Study design | No. of participants | Characteristics | Motivation | Satisfaction | Diagnosis | Treatment | Prognosis |
|---|---|---|---|---|---|---|---|---|---|---|
| Tattersall, 2009[52] | Cancer | Australia | Cross-sectional | 77 | X | X | X | | X | |
| Van Dalen, 2001[53] | Orthopaedics | Netherlands | Cross-sectional | 401-411/349* | X | X | | | | |
| Wieske, 2011[54] | Neurology | Netherlands | Cohort | 76 | | | X | | | |
| Wijers, 2010 [55] | Neurology | Netherlands | Cross-sectional | 183 | X | X | X | X | X | |

* indicates first doctors of patients who also participated in the study

## Appendix 2: Detailed Study findings

| Study | Participants | Study aim | Characteristics | Diagnosis/Treatment/Prognosis | Motivation/Satisfaction |
|---|---|---|---|---|---|
| Annandale, 1998[24] | Community sample of individuals | To explore doctor-patient disagreements, disagreement actions, and the relationship between them | | | **Motivation:** SO sought in 52.3% of diagnosis-related disagreements, 28.6% of prescribed drug-related disagreements, 53.3% of other treatment-related disagreements, 34.5% of disagreements where the patient felt the health problem had not been taken seriously, 33.3% of disagreements centred on the doctors' interactional style and 45.5% of other disagreements |
| Benson, 2001[25] | Patients seeking an SO at an eye hospital | To assess the value of patient initiated SOs for patients and third-party payers | 56% female, mean age 63 years, median age 66 years, 39% college-level education or higher, 39% employed, mean travel distance 42.5 miles, median travel distance 20 miles, 87% thought their insurer would pay | **Treatment:** 67.9% agreement with surgery recommendations, 41.7% agreement with laser treatment recommendations, 81.8% agreement with vitrectomy recommendations, and 100% agreement with scleral buckling procedures, cataract surgery and extruding scleral buckle removal. | **Motivation:** 41% sought an SO primarily because their first physician indicated that no treatment was possible or that even with treatment, the prognosis was poor. 20% wanted a better explanation of their problems, 9% specifically wanted a specialist from the hospital, 7% wanted an SO before surgery, 6% were not making progress |

| | | | | | |
|---|---|---|---|---|---|
| | | | for the SO | Major disagreement in 8.3% of cases for which no surgery had been recommended | with their current treatment, 6% were encouraged by a friend or family, 4% did not like their first physician, 3% wanted a specialist, 2% were encouraged by a family physician, and 2% believed that they were being pushed into treatment |
| Cecon. 2019[26] | Newly diagnosed breast cancer patients from 86 hospitals in Germany completed a postoperative mail survey | To examine breast cancer patients' reasons to seek a second opinion (SO) and the underlying variables. To find out more about the outcome of the SO, the perceived helpfulness and the effect on the physician-patient relationship. | | | Reasons to seek an SO were mostly unrelated to the physician-patient relationship. Reasons related to the physician-patient-relationship were associated with a lower education level.<br>A different treatment plan recommendation (25%) reportedly affected the patients' relationship with their primary physician. |
| Clauson, 2002[27] | Breast cancer patients (stage I, II or intraductal carcinoma) seeking a | To determine how often a SO on the local therapy of breast carcinoma changed patient | Mean age 51.4 years, 89% Caucasian, 70% more than a high school education, 80% | **Treatment:** The SO differed from the first opinion (FO) in 20.3% of cases | |

| | | | | | |
|---|---|---|---|---|---|
| | second surgical opinion at a breast centre | management, and to identify factors predictive of remaining at the SO site for therapy | employed outside the home | | |
| Fuchs, 2017 [28] | Cancer patients who participated in a series of lectures held by a regional cancer society on complementary and alternative medicine (CAM) | To explore cancer patients' motivation for seeking an SO | Males sought SOs more than females (79% males vs 53% females). Patients who reported low understanding of information sought an SO more often | **Treatment:** 66.7% of patients remained the same | **Motivation:** 80.6% wanted to check the correctness of treatment. 48.6% wanted to gain a better understanding of their diagnosis, with a positive correlation between this desire and experiencing a higher gain of information after an SO, and with this desire playing a stronger role in the decision to seek an SO in males than females<br>**Satisfaction:** 56.3% stated their trust in the attending physician was strengthened by getting an SO, with those patients feeling a high degree of satisfaction with the information about their planned treatment and the effects of the prescribed pharmaceuticals. 78.7% felt assured afterwards, with those patients feeling significantly less burdened by the disease |

| | | | | | |
|---|---|---|---|---|---|
| Gologorsky, 2013[29] | Patients that self-referred to an ophthalmology clinic seeking an SO | To determine the reasons that patients self-refer to an ophthalmology clinic seeking an SO | 57.5% female, mean age 60.9 years | | **Motivation:** 59.8% requested confirmation of diagnosis or more information, with 54% coming from outside ophthalmologists and 5.7% from outside optometrists. 40.2% had suffered a previous adverse experience with an outside medical provider, with 25.9% perceived treatment failure or complications, 6.9% poor provider communication skills, 4.6% distrust of provider and 2.9% poor bedside manner |
| Groß, 2017[30] | Newly diagnosed breast cancer patients with at least one postoperative histological finding of breast cancer, who underwent surgery in a breast cancer centre hospital | To examine the association between whether physicians discuss the possibility of seeking an SO with patients and the patients' decision to seek an SO, as well as the impact of seeking such an opinion on patients' trust in physicians | Patients informed about the possibility of requesting an SO, patients more actively involved in the decision-making process and patients with a school-leaving certificate were more likely to seek an SO. The better the information provided by doctors as reported by | | **Motivation:** Patients requesting an SO were more likely to not trust their physician. Patients aged between 18 and 66 years had less trust in their doctor than patients older than 75 years. The better the information provided by the doctor and the more patients were involved in the decision-making process, the higher the likelihood of patients indicating they had a trusting doctor-patient relationship |

| | | | | | |
|---|---|---|---|---|---|
| | | | patients, the lower the likelihood of seeking an SO | | |
| Katz, 2017[31] | Breast cancer patients (stage I, II or intraductal carcinoma) who had received surgery and had considered contralateral prophylactic mastectomy (CPM) with their first surgeon | To examine the association between patient report of first surgeon recommendation against CPM and the extent of discussion about it with 3 outcomes: patient satisfaction with surgery decisions, receipt of second opinion, and receipt of surgery by a second surgeon | SOs more common among patients who were younger, more educated, did not have Medicare health insurance and who worked for pay. Women who received a recommendation against CPM were not more likely to seek an SO (17.1% among patients with recommendation against CPM vs 15% among others) | | |
| Kurian, 2017[32] | Breast cancer patients (stage I, II or intraductal | To investigate the patterns and correlates of SO use, and their impact | Receiving a SO was significantly associated with a college education | | |

| | | | | | |
|---|---|---|---|---|---|
| | carcinoma) who had received surgery | on chemotherapy decisions and communication with oncologists | vs less education, a higher preference for making one's own treatment decisions vs a lower preference, and frequent use of internet-based support vs no use | | |
| Mellink, 2003[33] | Cancer patients seeking an SO at a surgical oncology outpatient clinic | To explore the sociodemographic and clinical characteristics of cancer patients seeking an SO consultation, and to analyse their SO-related motives, needs and expectations | 81.6% female, mean age 53 years, 50.5% less than a high school education | The mean score on information need was 3.4 about the disease, 3.7 about the treatment and 3.5 about the prognosis and expected course. Hope for a difference between the first and second opinion was expressed by 68% of the patients, whereas 22% hoped for identical advice | **Motivation:** With a range from 1 (not at all) to 4 (a lot), the mean score on internal motivation (associated with the need for reassurance and more certainty) was 3.66. The mean score on external motivation (related to negative experiences or unfulfilled needs) was 2.48. Externally motivated patients more often hoped for different advice. Patients with non-metastatic disease, a high level of anxiety disposition and preference for an active role in decision-making were relatively more often externally motivated. |

| | | | | | |
|---|---|---|---|---|---|
| Mellink, 2006[34] | Cancer patients seeking an SO at a surgical oncology outpatient clinic | To prospectively describe in a population of oncological SO patients the outcome of routine revisions of histopathological and radiological material, the frequency and extent of discrepancy between the second and first opinion, and the location of further treatment or follow-up | 87.3% female, mean age 52 years | **Diagnosis/Treatment/Prognosis:** Major difference in diagnosis, treatment or prognosis was identified in 16.4% of patients, minor difference in 15.5% and no difference in 68.1%. Pathology review resulted in a difference which affected prognosis or therapy in 3.4% of cases and a difference not affecting prognosis or therapy in 2.8%. Radiology review resulted in a difference affecting prognosis or therapy in 1.6% of cases and a difference not affecting prognosis or therapy in 2.8% | |
| Meyer, 2015[35] | Patients who sought an SO whilst enrolled in a national SO program allowing employee-beneficiaries to request free SOs | To examine the outcomes of SOs provided by a national patient-initiated SO program | | **Diagnosis:** 56.8% cases confirmed, 17% clarified, and 14.8% changed. Anaesthesiology, gastroenterology, neurology, and rheumatology resulted in significantly more changes than average. Cardiovascular disease, | **Motivation:** 41.3% needed help choosing treatment options, 22.5% had symptoms that were not improving, 18% were questioning whether to proceed with recommended surgery, 6.3% sought a diagnosis, 6% did not understand their diagnosis, and 6% were sceptical of their |

| | | | | | |
|---|---|---|---|---|---|
| | from expert specialists | | | medical oncology and haematology, surgical oncology, and urology resulted in significantly fewer. Minor clinical impact in 46.3% of cases, moderate impact in 18.2% of cases, and major impact in 2.7% of cases. Critical care/pulmonary medicine, gastroenterology, infectious diseases, neurology, and obstetrics and gynaecology resulted in significantly more estimates of moderate/major clinical impact than average. General surgery, ophthalmology, and radiation oncology resulted in significantly fewer<br>**Treatment:** 26.4% cases confirmed, 26.9% clarified, and 37.4% changed. Allergy and immunology, anaesthesiology, gastroenterology, neurological surgery, obstetrics and | physician<br>**Satisfaction:** 94.7% were satisfied with the SO experience, 89.6% had their questions answered and 87.3% were more confident in their diagnosis or treatment choice afterwards |

| | | | | | |
|---|---|---|---|---|---|
| | | | | gynaecology, otolaryngology, physical medicine and rehabilitation, and rheumatology resulted in significantly more changes than average. General surgery, medical oncology and haematology, surgical oncology, and urology resulted in significantly fewer. Minor clinical impact in 50.1% of cases, moderate impact in 26.5% of cases, and major impact in 4.2% of cases. Colon and rectal surgery, medical oncology and haematology, obstetrics and gynaecology, and thoracic surgery resulted in significantly more estimates of moderate/major impact than average. Cardiovascular disease, general surgery, internal medicine, neurology, ophthalmology, and physical medicine and | |

| | | | | | |
|---|---|---|---|---|---|
| | | | | rehabilitation resulted in significantly fewer **Diagnosis/Treatment:** 10.6% of cases had changes in both diagnosis and treatment | |
| Mordechai, 2015[36] | Parents of children with cancer recently treated in a paediatric haematology oncology department | To investigate the epidemiology and motivations of the families who sought an SO | More common for those with a higher socioeconomic status, those with a higher number of educational years and those more non-religious | **Treatment:** 35.1% were advised to change their therapy | **Satisfaction:** 56.7% were satisfied with the second opinion, 29.7% found it was not effective and 24.3% found it unsettling |
| Morrow, 2009[37] | Breast cancer patients (stage I, II or intraductal carcinoma) | To evaluate the association of patient-reported initial recommendations by surgeons and those given if an SO was sought with receipt of initial mastectomy, and to assess the use of | **Characteristics:** More common for woman with a higher educational level and those initially advised to undergo mastectomy | **Treatment:** 12.1% received a discordant opinion from a second surgeon. 20.2% of patients who received an initial mastectomy recommendation received an SO for BCS. 11.9% of patients who received an initial BCS recommendation received an SO for mastectomy. 56.5% of patients | |

| | | | | | |
|---|---|---|---|---|---|
| | | mastectomy after attempted breast-conserving surgery (BCS) | | who did not receive a first surgeon's recommendation similarly did not receive one from a second surgeon | |
| Mustafa, 2002 [38] | Patients with chronic unresolved symptoms or treatment issues seeking an SO in a general medicine outpatient clinic | To explore the nature and possible benefits of patient-generated SOs in general internal medicine | 58.2% female, mean age 46 years | **Diagnosis:** When an SO was sought for diagnostic reasons, a definite diagnosis was established in only 10% of cases<br>**Treatment:** When an SO was sought for therapeutic advice, a useful new treatment plan was obtained in 71% of cases | **Motivation:** 84.6% had poorly defined complaints that could not be satisfactorily explained or diagnosed by their original physician, and 15.4% sought management advice (3% sought better control of their blood pressure and 1.5% sought better control of their diabetes) |
| Okamoto, 2013[39] | Patients in the SO clinic (group A) and general patient waiting area (group B) of a university hospital | To investigate the characteristics and motivation of patients who seek SOs in Japan's universal healthcare system, and to explore how these SOs affect understanding and management | 51.7% female, 56.2% 40-64 years and 70.5% no medical provider in the family. 54.1% had a 4-year college education or higher. Those who finished graduate school were 9.5 times, and those who completed 4-year college were 2.1 times more likely to | **Diagnosis/Treatment:** 8.8% of SOs were the same, 41.5% were almost the same, 27.2% were partially different, and 12.9% were different<br>**Treatment:** 17.7% of patients would not ask to change their treatment plan as a result of the SO, 10.2% would be unlikely to ask, 23.1% would ask to partially change, and 22.4% would ask to | **Motivation (group A):** 100% believed an SO would be sought for better understanding, 97% believed for decision-making, 77.6% believed for changing ongoing treatment, and 50.7% believed for changing doctor<br>**Motivation (group B):** 92.6% believed an SO would be sought for better understanding, 95.1% believed for decision-making, 84.1% believed for changing ongoing treatment, and 67.9% |

| | | | | | |
|---|---|---|---|---|---|
| | | | obtain an SO than those with a high school education or lower | change**.** When the SO was the same or almost the same as the FO, 25.7% would seek a change or partial change. When the SO and FO differed, 67.8% would seek a change or partial change | believed for changing doctor<br>**Satisfaction (group A):** 92.5% better understood treatment options, 87.9% better understood their illness and plan, 87.7% better understood that their treatment was specifically designed for their health condition, 81.8% better understood the risks of their treatment, and 81.5% better understood uncertainty in medicine<br>**Satisfaction (group B):** 81.5% better understood treatment options, 77.8% better understood the risks of their treatment, 73.2% better understood their illness and plan, 66.7% better understood that their treatment was specifically designed for their health condition, and 61.3% better understood uncertainty in medicine |
| Philip, 2010[40] | Advanced cancer patients attending specialist clinics in a | To explore the views on SOs held by advanced cancer patients and their | **According to group B:** 84% characterised SO patients (SOPs) as | | **Motivation (group A):** 26.8% of reasons given related to concerns around communication, 32.1% related to the |

| | | | | | |
|---|---|---|---|---|---|
| | quaternary hospital (group A) and their FO medical oncologists (group B) | medical oncologists, including motivation, satisfaction and the impact they may have on the doctor-patient relationship | having greater information needs, 58% believed they had greater psychosocial needs, and 77% believed they took more physician time and energy than the overall patient population | | extreme and desperate nature of the situation, 12.5% related to the need for reassurance, 12.5% related to concerns with care, and 8.9% related to SOs being prompted by other parties including family, friends or as result of information in the media<br>**Motivation (according to group B):** 75% of reasons suggested related to a need for additional information, 70% related to family or friends urging an SO, 70% related to a need for reassurance regarding diagnosis and treatment course, 60% related to a need for communication in a different form, 60% related to a need for information in a different form, 53% related to a need to leave 'no stone unturned', 51% related to a need for different style or personality in the doctor, 48% related to the patient requesting more treatment, 37% related to reassurance offered by the public status of the doctor or their |

| | | | | | |
|---|---|---|---|---|---|
| | | | | | institution, and 14% related to an SO being akin to getting quotes<br>**Satisfaction (group A):** 94.1% found the SO helpful, with 44.2% of responses relating satisfaction to the communication or manner of the second doctor and 38.4% relating satisfaction to reassurance<br>**Satisfaction (according to group B):** 91% considered reassurance to be important to patient satisfaction, 83% suggested different or more comprehensive information, 83% suggested an approach to care which was more commensurate with the patient's needs, 69% suggested the skills of the doctor, 57% suggested the different organisation of time within the consultation, and 51% suggested the status of the doctor to be important |
| Radhakrishnan, 2017[41] | Newly diagnosed local-stage prostate | To assess the frequency of and reasons for SOs for local-stage prostate | Younger men and men with college-level education or higher were | | **Motivation:** 50.8% wanted more information about their cancer (younger men and men with a college-level |

| | | | | | |
|---|---|---|---|---|---|
| | cancer patients | cancer and the characteristics of the patients who seek them, and to assess whether SOs are associated with treatment choice and perceived quality of prostate cancer care | more likely to obtain an SO | | education or higher more likely to), 46.3% wanted to be seen by the best doctor (younger men more likely to), 31% were encouraged by a family member or friend to obtain an SO, 25% wanted to find out about treatment not offered by their first doctor, and 15.5% were dissatisfied with their initial urologist (patients aged 75 years or older least likely to) |
| Ramsey, 2011[42] | Newly diagnosed local-stage prostate cancer patients and their urologists at academic urology clinics | To compare patient preferences and urologist recommendations for treatment among local-stage prostate cancer patients presenting for initial management consultations versus SOs | Men seeking SOs were significantly younger, more educated, more likely to have private insurance and more likely to be employed. 53.8% had low-risk disease and 23.1% listed two or more non-cancer comorbidities at diagnosis | **Treatment:** Prostatectomy was the dominant treatment recommended at SO visits, with less than 20% of urologists reporting recommending other options. During initial consultations, other treatments were more likely to be recommended in addition to prostatectomy. SO consultations associated with a fewer number of treatment recommendations (0.52 fewer) | |

| | | | | | |
|---|---|---|---|---|---|
| Sato, 1999[43] | Primary care patients in the general medicine clinic of a university hospital | To describe the sociodemographic characteristics of SO patients and to determine the factors related to this behaviour | 60.5% female, mean age 45.2 years, 62.6% married, 88.3% employed. SOPs had a significantly higher educational level than doctor-shopping patients (DSPs), with 78.8% having more than nine years of education. General Health Questionnaire (GHQ) scores of SOPs were significantly higher than those of first opinion patients (FOPs), suggesting that SOPs are more anxious. Compared with FOPs, SOPs were more likely to have a chronic illness and to believe they were in poor health, also taking | | |

| | | | | | |
|---|---|---|---|---|---|
| | | | more advice from anybody. DSPs were more likely than SOPs to believe they were in poor health, to mistrust their diagnosis and treatment, and to have high expectations for the hospital. FOPs had a significantly higher frequency of diagnosis for endocrinological and metabolic disorders than did SOPs. SOPs had a significantly higher frequency of diagnosis for obstetric and gynaecological disorders than did DSPs. DSPs had the most frequent diagnosis of psychiatric illnesses compared with FOPs and SOPs | | |

| | | | | | |
|---|---|---|---|---|---|
| Schook, 2014[44] | Lung cancer patients referred to a specialist pulmonary oncology outpatient clinic | To collect data from the initial evaluation of patients referred to the clinic for an SO and compare these with the data of the SO conducted to identify discrepancies in diagnosis, stage and therapeutic advice | 57.4% male, mean age 59 years | **Diagnosis/Treatment:** There were discrepancies for 9% of diagnosis, 12.8% of stage, and 37.2% of therapeutic advice. 58.2% of patients with discrepancies had a potential major impact on patient outcomes, 21.9% had a potential minor impact and 19.8% had a potential identical impact | |
| Shmueli, 2016[12] | Active members of the largest regional health fund who visited at least one specialist within a 20-month period (group A) plus a representative random sample of the general adult population (group B) | To estimate how many people seek SOs and to determine the characteristics of SO seekers | **Group A:** More women than men, native-born and established immigrants than recent immigrants, older people than younger people, people in high and middle socio-economic levels than low income level, people living in central areas and intermediate localities than people living in | | |

| | | | | | |
|---|---|---|---|---|---|
| | | | peripheral areas, and people with chronic conditions than people with no chronic conditions<br>**Group B:** More women than men, native-born and established immigrants than recent immigrants, and people living in central areas and intermediate localities than people living in peripheral areas. Those who perceived their health as good or very good sought fewer SOs than those who perceived their health as not so good | | |

| | | | | | |
|---|---|---|---|---|---|
| Shmueli, 2017[45] | Representative random sample of the general adult population | To characterise SO seekers, their reasons for seeking an SO and choosing a specific physician, and their perceived outcomes following the SO | | **Diagnosis/Treatment:** 56.1% mentioned there was a difference in diagnosis or treatment between the FO and SO | **Motivation:** 38.1% stated they wanted to verify their diagnosis with another doctor or they had doubts about the treatment recommended, 19.4% were seeking a sub-specialist's opinion, 19.2% were dissatisfied with communication with their first doctor or felt they didn't receive enough information, and 15.4% believed previous treatments were ineffective<br>**Satisfaction:** 84.3% were satisfied with the SO, 91% preferred the SO over the FO and 76.5% experienced health improvement after the SO |
| Shmueli. 2019 [46] | Patients aged 21 years and above who visited at least one specialist over an 18 months period, either in the secondary care or privately via the | To evaluate the utilization (overall and by specialty) and the characteristics of second-opinion seekers by insurance type (either health fund or | second-opinion seekers via the health fund tended to be females, of age 40–59 years and with chronic conditions. In contrast, second-opinion seekers via the supplementary insurance tended to be native-born | | |

| | | | | | |
|---|---|---|---|---|---|
| | supplementary insurance | supplementary insurance) in a mixed private-public | and established immigrants, in a high socioeconomic level and living in central areas. | | |
| Shmueli. 2019a[47] | Representative random sample of the general adult population | To evaluate (1) the characteristics of people seeking SOs in the private system vs. the public system in Israel; (2) the reasons for seeking private SOs; and (3) the perceived outcomes of SOs given in a private system vs. a public system | Low self-reported income group, immigrants (immigrated to Israel after 1989) and religious people tended to seek SOs from the public system more than others. | | The main reason for seeking a SO from a private physician rather than from the public system was an assumption that private physicians are more professional (n = 58, 45.7% of 127). The other reasons were prior acquaintance with the physician or a word-of-mouth about the specific private physician (n = 21, 16.5%), waiting time at the health fund (n = 18, 14.2%), that private physicians have better attitudes (n = 13, 10.2%) or other reasons such as flexible hours, restrictions of the public health fund, etc. |
| Sutherland. 1989 [48] | Patients referred for the first time to a university-based gastrointestinal unit. | To investigate how many of the patients, referred for the first time, were seeking a second opinion- | Patients who sought a second opinion were more apt to have symptoms for >2 years, | | Main reasons for seeking a SO These patients either (a) believed that the original gastroenterologist had not spent |

| | | | | | |
|---|---|---|---|---|---|
| | | -i.e., a second consultation within 2 years of seeing a gastroenterologist. | perceive their health to be fair to poor, fewer than two have seen general practitioners in the past year, have spent >6 days in hospital in the last year. | | enough time with them or (b) wanted a confirmation of the original diagnosis. |
| Sutherland. 1994 [49] | Patients attending a university-based gastroenterology clinic were asked to complete a self-administered questionnaire. | To determine psychosocial determinants of two measures of health care use: seeking a second opinion and alternative medicine use, and to assess whether changes in these two measures of health care use had taken place during the past 4 to 5 years. | Seeking a second opinion was negatively related to internal locus of control, perceived health status, and demanding to know all details of treatment. Eight percent (28) of all patients saw an alternative practitioner for the same problem for which they saw the gastroenterologist compared to 9% 4 years ago. | | |

| | | | | | |
|---|---|---|---|---|---|
| Tam, 2005[50] | Gynaecologic cancer patients attending a gynaecologic cancer clinic of a tertiary referral (TR) centre | To determine the prevalence and predictors for seeking an SO and the utilisation of CAM among gynaecologic cancer patients, and the relationship between the two behaviours | Mean age 48.7 years. More likely to seek an SO if late-stage disease, previous treatment with radiotherapy, tertiary education or income >US$30,000. 71.3% had used CAM, being 2.47 times more likely than non-SO seekers | | **Motivation:** 45.7% of patients had complications or side effects arising from the standard cancer treatment, 37% just wanted to see more doctors, and 17.4% wanted some advice to maintain a better 'well-being'. Patients who gave a positive answer to 'I am doubtful to what my doctors have done on me' or 'I would receive better care if I see more doctors' were more likely to seek an SO |
| Tan, 2014[51] | Women seeking an SO for management of symptomatic uterine fibroids at a multidisciplinary uterine fibroid treatment centre in a tertiary care facility | To describe the early experience of a comprehensive uterine fibroid treatment centre and report results in women seeking an SO for management of symptomatic uterine fibroids | Mean age 43.8 years, 79% had not had prior therapy | **Diagnosis:** Nearly all had received a diagnosis of uterine fibroids from outside clinics but only 86.8% were found to have them<br>**Treatment:** Most had been offered hysterectomy from outside facilities. Medical therapy or no further treatment was recommended for 22% of | |

| | | | | | |
|---|---|---|---|---|---|
| | | | | patients. 77.9% underwent procedures and 7.3% underwent hysterectomy. 53.2% elected to transfer their care to the treatment centre | |
| Tattersall, 2009[52] | Cancer patients seeking an SO in a medical oncology clinic | To investigate the frequency, goals and outcomes of SO consultations in a medical oncology clinic | **Characteristics:** 76.6% female, median age 55 years, 68.8% only English spoken at home, 85.7% married, 89.6% no medical or allied health training, 33.8% had a university degree, 68.8% had started treatment recommended by their first oncologist more and 0% believed less, 35.3% believed SO doctor seemed more knowledgeable and 2% believed less, 51% believed SO doctor answered concerns and | | **Motivation:** 70.1% required more information about treatment options or decisions, 61% sought reassurance that diagnosis or treatment already suggested was appropriate, 32.5% required more information about their cancer, and 31.2% were dissatisfied with the level of information or communication received so far<br>**Satisfaction:** 39.2% believed SO doctor listened |

| | | | | | |
|---|---|---|---|---|---|
| | | | 0% believed they did not, 52.9% believed SO doctor gave them more confidence and 7.8% believed less, 47.1% believed SO doctor made them feel more confident and 3.9% believed less, 41.2% believed SO doctor was more friendly and 0% believed less, 49% believed they received more information from SO doctor and 2% believed less, and 51% believed they received new information from SO doctor<br>**Treatment:** 41.6% of patients intended to change treatment, with 28.6% continuing with | | |

| | | | | | |
|---|---|---|---|---|---|
| | | | their second oncologist to do this and 13% staying with their first oncologist. 9.1% of patients intended to continue with their second oncologist but not change their treatment, giving 37.7% of patients who intended to change their oncologist | | |
| Van Dalen, 2001[53] | New patients at an orthopaedic surgical outpatient clinic at a university hospital seeking an SO (group A) and their first opinion consultants (group B) | To identify the characteristics, motivating factors and first consultant experiences of patients who seek second orthopaedic surgical outpatient opinions | **Characteristics:** Those who felt their relationship with their FO consultant was poorer, those whose FO consultant practiced nearer to the SO centre and those that visited an FO consultant working in a larger group of consultants had a higher | | **Motivation (group A):** 84% patient wanted more information about treatment possibilities, 67% patient wanted more information about the condition, 61% FO consultant had no solution to the problem, 60% results of treatment were disappointing, 51% patient dissatisfied with FO consultant, 43% FO consultant offered no treatment, 40% patient believed the diagnosis was incorrect, 39% patient had no confidence |

| | | | | | |
|---|---|---|---|---|---|
| | | | likelihood of seeking an SO on their own initiative | | in the FO consultant, 37% FO consultant found no substantive diagnosis, 26% patient hoped to get a different diagnosis, 16% family/friends had had good experience with a certain consultant, 12% patient disapproved of the recommended treatment, 9% patient was concerned about the diagnosis, and 8% family/friends had had good experience with a certain treatment<br>**Motivation (according to group B):** 33% suggested the results of the treatment had been disappointing, 28% suggested the patient wanted more information about the treatment, 16% suggested the patient wanted more information about the condition, 7% suggested the patient disapproved of the recommended treatment, 3% suggested the patient was concerned about the diagnosis, and 2% suggested there were communication problems |

| | | | | | |
|---|---|---|---|---|---|
| Wieske, 2011[54] | Patients referred to the neurological day-care clinic of an academic medical centre for an SO or TR | To investigate long-term patient satisfaction with a day-care admission for a neurological SO or TR | | | **Satisfaction:** Decrease in satisfaction shown on visual analogue scale (VAS) ranging from 0 'not at all' to 10 'completely' when comparing level of satisfaction with referring physician and 2 years after SO (5.4 vs 5.3; -0.1) and when comparing level of satisfaction directly after SO and 2 years after SO (-2.6) |
| Wijers, 2010 [55] | Patients referred to the neurological day-care clinic of an academic medical centre for an SO or TR | To explore the expectations of patients who seek a neurological SO or TR, and to assess patient satisfaction with a day-care admission for such a consultation | Mean age 47 years, 55.2% female, median duration of symptoms 2 years | **Diagnosis/Treatment:** 56% received a new diagnosis and/or treatment advice | **Motivation:** 59% expected a new diagnosis or treatment, 28% expected an explanation, and 6% expected confirmation of their diagnosis or treatment<br>**Satisfaction:** Overall satisfaction with SO 7.4 on VAS ranging from 0 'not at all' to 10 'completely' compared to 5.5 with FO. Higher scores for SO than FO with all aspects of satisfaction (own involvement in the conversation, physician's information giving, own involvement in decision-making, physicians' emotional support, and |

| | | | | | |
|---|---|---|---|---|---|
| | | | | | general satisfaction). Patients who received a new diagnosis/treatment were equally as satisfied with the consultation as patients who did not (7.5 vs 7.4) |

## Appendix 3: Risk of bias assessment

| Study | 1 | 2 | 3 | 4 | 5 | 6 | 7 | 8 | 9 | 10 | 11 | 12 | 13 | 14 |
|---|---|---|---|---|---|---|---|---|---|---|---|---|---|---|
| Annandale, 1998 [24] | Y | Y | NR | Y | N | N | NA | NA | Y | NA | Y | N | NA | N |
| Benson, 2001 [25] | Y | Y | NR | Y | N | N | NA | NA | Y | NA | Y | N | NA | N |
| Cecon, 2019 [26] | Y | Y | Y | Y | N | N | NA | NA | Y | NA | Y | N | NA | N |
| Clauson, 2002 [27] | Y | Y | NR | Y | Y | N | NA | NA | Y | NA | Y | N | NA | N |
| Fuchs, 2017 [28] | Y | Y | N | Y | N | N | NA | NA | Y | NA | Y | N | NA | N |
| Gologorsky, 2013 [29] | Y | Y | Y | Y | N | N | NA | NA | Y | NA | Y | N | NA | N |
| Groß, 2017 [30] | Y | Y | Y | Y | Y | N | NA | NA | Y | NA | Y | N | NA | Y |
| Katz, 2017 [31] | Y | Y | Y | Y | N | N | NA | NA | Y | NA | Y | N | NA | Y |
| Kurian, 2017 [32] | Y | Y | Y | Y | Y | N | NA | NA | Y | NA | Y | N | NA | Y |
| Mellink, 2003 [33] | Y | Y | Y | Y | Y | N | NA | NA | Y | NA | Y | N | NA | N |
| Mellink, 2006 [34] | Y | Y | Y | Y | Y | Y | NA | NA | Y | NA | Y | N | Y | N |
| Meyer, 2015 [35] | Y | Y | Y | Y | Y | N | NA | NA | Y | NA | Y | N | NA | N |
| Mordechai, 2015 [36] | Y | Y | Y | Y | Y | N | NA | NA | Y | NA | Y | N | NA | N |
| Morrow, 2009 [37] | Y | Y | Y | Y | Y | N | NA | NA | Y | NA | Y | N | NA | N |
| Mustafa, 2002 [38] | Y | Y | Y | Y | N | N | NA | NA | Y | NA | Y | N | NA | N |

| Study | 1 | 2 | 3 | 4 | 5 | 6 | 7 | 8 | 9 | 10 | 11 | 12 | 13 | 14 |
|---|---|---|---|---|---|---|---|---|---|---|---|---|---|---|
| Okamoto, 2013 [39] | Y | Y | Y | Y | N | N | NA | NA | Y | NA | Y | N | NA | N |
| Philip, 2010 [40] | Y | Y | Y | Y | N | N | NA | NA | Y | NA | Y | N | NA | N |
| Radhakrishnan, 2017 [41] | Y | Y | Y | Y | Y | N | NA | NA | Y | NA | Y | N | NA | Y |
| Ramsey, 2011 [42] | Y | Y | Y | Y | Y | Y | NA | NA | Y | NA | Y | N | Y | Y |
| Sato, 1999 [43] | Y | Y | Y | Y | Y | N | NA | NA | Y | NA | Y | N | NA | Y |
| Schook, 2014 [44] | Y | Y | Y | Y | Y | N | NA | NA | Y | NA | Y | N | NA | N |
| Shmueli, 2016 [12] | Y | Y | Y | Y | Y | N | NA | NA | Y | NA | Y | N | NA | Y |
| Shmueli, 2017 [45] | Y | Y | Y | Y | Y | N | NA | NA | Y | NA | Y | N | NA | N |
| Shmueli, 2019 [46] | Y | Y | Y | Y | N | N | NA | NA | Y | NA | Y | N | NA | Y |
| Shmueli, 2019a [47] | Y | Y | Y | Y | Y | N | NA | NA | Y | NA | Y | N | NA | N |
| Sutherland. 1989 [48] | Y | Y | Y | Y | N | N | NA | NA | Y | NA | Y | N | NA | Y |
| Sutherland. 1994 [49] | Y | Y | Y | Y | N | N | NA | NA | Y | NA | Y | N | NA | Y |
| Tam, 2014 [51] | Y | Y | Y | Y | Y | N | NA | NA | Y | NA | Y | N | NA | N |
| Tam, 2005 [50] | Y | Y | Y | Y | Y | N | NA | NA | Y | NA | Y | N | NA | Y |
| Tattersall, 2009 [52] | Y | Y | Y | Y | N | N | NA | NA | Y | NA | Y | N | NA | N |
| Van Dalen, 2001 [53] | Y | Y | Y | Y | Y | N | NA | NA | Y | NA | Y | N | NA | Y |

| Study | 1 | 2 | 3 | 4 | 5 | 6 | 7 | 8 | 9 | 10 | 11 | 12 | 13 | 14 |
|---|---|---|---|---|---|---|---|---|---|---|---|---|---|---|
| Wieske, 2011 [54] | Y | Y | N | Y | Y | Y | NA | NA | Y | NA | Y | N | N | Y |
| Wijers, 2010 [55] | Y | Y | Y | Y | Y | N | NA | NA | Y | NA | Y | N | NA | Y |

Y, Yes; N, No; NR, Not Reported; NA, Not Applicable

***Study quality assessment***

Study quality was assessed using the NIH Quality Assessment Tool for Observational Cohort and Cross-Sectional Studies.[34] The following 14 questions were answered for each study:

1. Was the research question or objective in this paper clearly stated?

2. Was the study population clearly specified and defined?

3. Was the participation rate of eligible persons at least 50%?

4. Were all the subjects selected or recruited from the same or similar populations (including the same time period)? Were inclusion and exclusion criteria for being in the

study prespecified and applied uniformly to all participants?

5. Was a sample size justification, power description, or variance and effect estimates provided?

6. For the analyses in this paper, were the exposure(s) of interest measured prior to the outcome(s) being measured?

7. Was the timeframe sufficient so that one could reasonably expect to see an association between exposure and outcome if it existed?

8. For exposures that can vary in amount or level, did the study examine different levels of the exposure as related to the outcome (e.g., categories of exposure, or exposure measured as continuous variable)?

BMJ Publishing Group Limited (BMJ) disclaims all liability and responsibility arising from any reliance placed on this supplemental material which has been supplied by the author(s)


9. Were the exposure measures (independent variables) clearly defined, valid, reliable, and implemented consistently across all study participants?

10. Was the exposure(s) assessed more than once over time?

11. Were the outcome measures (dependent variables) clearly defined, valid, reliable, and implemented consistently across all study participants?

12. Were the outcome assessors blinded to the exposure status of participants?

13. Was loss to follow-up after baseline 20% or less?

14. Were key potential confounding variables measured and adjusted statistically for their impact on the relationship between exposure(s) and outcome(s)?
