# Supplementary material for: Patient-initiated second medical consultations—patient characteristics and motivating factors, impact on care and satisfaction: a systematic review
Source: BMJ Open. 2021 Sep 24;11(9):e044033. doi: 10.1136/bmjopen-2020-044033 (PMC8475134; doi:10.1136/bmjopen-2020-044033)
Supplement: Author's manuscript [file bmjopen-2020-044033.draft_revisions.pdf]

BMJ Open

BMJ Open is committed to open peer review. As part of this commitment we make the peer review history of every article we publish publicly available.

When an article is published we post the peer reviewers' comments and the authors' responses online. We also post the versions of the paper that were used during peer review. These are the versions that the peer review comments apply to.

The versions of the paper that follow are the versions that were submitted during the peer review process. They are not the versions of record or the final published versions. They should not be cited or distributed as the published version of this manuscript.

BMJ Open is an open access journal and the full, final, typeset and author-corrected version of record of the manuscript is available on our site with no access controls, subscription charges or pay-per-view fees (http://bmjopen.bmj.com).

If you have any questions on BMJ Open's open peer review process please email info.bmjopen@bmj.com

BMJ Open

Patient-initiated second medical consultations: patient characteristics and motivating factors, impact on care, satisfaction and cost-effectiveness: A systematic review

Journal:	*BMJ Open*
Manuscript ID	bmjopen-2020-044033
Article Type:	Original research
Date Submitted by the Author:	07-Sep-2020
Complete List of Authors:	Greenfield, Geva; Imperial College London Department of Life Sciences, Department of Primary Care and Public Health, School of Public Health<br>Shmueli, Liora; Ben-Gurion University of the Negev, Department of Health Systems Management; Tel Aviv University - Recanati Graduate School of Business Administration ,<br>Harvey, Amy; Imperial College London School of Public Health<br>Quezada-Yamamoto, Harumi; Imperial College London, Department of Primary Care and Public Health<br>Davidovitch, Nadav; Ben-Gurion University of the Negev, Department of Health Systems Management<br>Pliskin, Joseph ; Ben-Gurion University of the Negev<br>Rawaf, Salman; Imperial College London, Primary care and Public Health<br>Majeed, Azeem; Imperial College, Primary Care<br>Hayhoe, Benedict; Imperial College London School of Public Health
Keywords:	GENERAL MEDICINE (see Internal Medicine), HEALTH ECONOMICS, HEALTH SERVICES ADMINISTRATION & MANAGEMENT, Quality in health care < HEALTH SERVICES ADMINISTRATION & MANAGEMENT

SCHOLARONE™
Manuscripts

I, the Submitting Author has the right to grant and does grant on behalf of all authors of the Work (as defined in the below author licence), an exclusive licence and/or a non-exclusive licence for contributions from authors who are: i) UK Crown employees; ii) where BMJ has agreed a CC-BY licence shall apply, and/or iii) in accordance with the terms applicable for US Federal Government officers or employees acting as part of their official duties; on a worldwide, perpetual, irrevocable, royalty-free basis to BMJ Publishing Group Ltd ("BMJ") its licensees and where the relevant Journal is co-owned by BMJ to the co-owners of the Journal, to publish the Work in this journal and any other BMJ products and to exploit all rights, as set out in our licence.

The Submitting Author accepts and understands that any supply made under these terms is made by BMJ to the Submitting Author unless you are acting as an employee on behalf of your employer or a postgraduate student of an affiliated institution which is paying any applicable article publishing charge ("APC") for Open Access articles. Where the Submitting Author wishes to make the Work available on an Open Access basis (and intends to pay the relevant APC), the terms of reuse of such Open Access shall be governed by a Creative Commons licence – details of these licences and which Creative Commons licence will apply to this Work are set out in our licence referred to above.

Other than as permitted in any relevant BMJ Author's Self Archiving Policies, I confirm this Work has not been accepted for publication elsewhere, is not being considered for publication elsewhere and does not duplicate material already published. I confirm all authors consent to publication of this Work and authorise the granting of this licence.

For peer review only

Patient-initiated second medical consultations: patient characteristics and motivating factors, impact on care, satisfaction and cost-effectiveness: A systematic review

Geva Greenfield[1], Liora Shmueli[2], Amy Harvey[1], Harumi Quezada-Yamamoto[1], Davidovitch[3], Joseph Pliskin[3], Salman Rawaf[1], Azeem Majeed[1], Benedict Hayhoe[1]

[1] Department of Primary Care and Public Health, School of Public Health, Imperial College London, UK

[2] Department of Management, Bar Ilan University, Ramat Gan, Israel

[3] Department of Health Systems Management and Department of Industrial Engineering & Management, Ben-Gurion University of the Negev, Beer-Sheva, Israel

*** Corresponding author**: Geva Greenfield, Department of Primary Care and Public Health, School of Public Health, Imperial College London, 314 The Reynolds Building, St Dunstan's Road, London W6 8RP, g.greenfield@ic.ac.uk

The manuscript includes 2,995 words, 56 references, 1 figure, and 2 tables. The abstract includes 291 words.

For peer review only

Abstract

Objectives: To review the characteristics and motivations of patients seeking second opinions, and the impact of such opinions on patient management, satisfaction, and cost-effectiveness.

Data sources: Embase, Medline, PsycINFO and HMIC databases.

Study design: A systematic literature search was performed for terms related to second opinion and patient characteristics. Study quality was assessed using the National Institutes of Health (NIH) Quality Assessment Tool for Observational Cohort and Cross-Sectional Studies.

Data collection / Extraction methods: We included articles focused on patient-initiated second opinions, which provided quantitative data on their impact on diagnosis, treatment, prognosis or patient satisfaction, described the characteristics or motivating factors of patients who initiated a second opinion, or the cost-effectiveness of patient-initiated second opinions.

Principal findings: Thirty-one articles were included in the review. 27 studies considered patient characteristics, 18 patient motivating factors, 10 patient satisfaction, and 17 clinical agreement between the first and second opinion. Seeking a second opinion was more common in women, middle age patients, more educated patients; and in people having a chronic condition, with higher income or socioeconomic status or living in central urban areas. Patients seeking a second opinion sought to gain more information or reassurance about their diagnosis or treatment. While many second opinions confirm the original diagnosis or treatment, discrepancies in opinions had a potential major impact on patient outcomes in up to 58.2% of cases. No studies reporting on the cost-effectiveness of patient initiated second opinions.

Conclusions: Research is needed to examine cost-effectiveness of second opinions and to identify patient groups that are likely to benefit from a second opinion. In the context of rising pressure on primary and secondary care services, it is important to set up clear mechanisms for patients seeking second opinions in both public and private systems.

Keywords: Second opinion, decision making, diagnostic discrepancies, cost-effectiveness, help-seeking behaviours.

For peer review only

Strengths and limitations of this study

- The review provides an up-to-date summary of the scientific literature on patient-initiated second medical consultations.
- Seeking a second opinion was more common in women, middle age patients, more educated patients; and in people having a chronic condition, with higher income or socioeconomic status or living in central urban areas. Second opinions were sought to gain more information or reassurance about diagnosis or treatment. Second opinions often confirmed the first opinion, however discrepancies in opinions had a potential major impact on patient outcomes.
- There is challenging in interpreting findings in this review, consisting of studies from different countries and different healthcare systems, where different insurance models are in place.

For peer review only

Background

A second medical opinion (SO) is a medical decision-making tool for patients, physicians, hospitals and insurers. For patients, it is a way to gain an additional opinion on a diagnosis, treatment or prognosis from another physician [1]. Physicians seeking another colleague's opinion may refer a patient to gain further advice (consultant to consultant referrals). Many health insurers mandate SO programs to reduce medical costs and eliminate ineffective or sub-optimal treatments [2,3]. Hospitals may also require second reviews as part of routine pathology, radiology reviews or for legal purposes. consultant to consultant referrals. Patients in primary care may also request an opinion from a second specialist when unhappy with the opinion from the first specialist.

The clinical impact of insurer-initiated or hospital-initiated second reviews on diagnosis is well documented [4–8]. The value of SOs in pathology and radiology is also well documented, with improvements in the quality of care and reductions in the rate of diagnostic error firmly established[5–8]. The cost-effectiveness of routine and mandatory SO programs has similarly been extensively studied [2,9]. However, the cost-effectiveness of patient-initiated SOs, and the reasons for initiating SOs, currently remain unclear.

As many patients seek a SO before committing to a treatment plan or a surgery, it is important to understand the advantages vs disadvantages of patient-initiated SOs for themselves, physicians, health services and insurers [10–12]. Seeking a SO may benefit patients medically, provided that the SO is of equal or better quality than the first opinion (FO) [13]. Diagnostic errors, thought to occur in 10% to 15% of cases in general medicine, may be reduced as a result, and better treatment may be recommended [14–16]. SOs may also benefit patients psychologically by enabling them take control of their care and by offering reassurance [17]. However, it is possible that many SOs do not yield medical benefits for patients and may critically delay the treatment [13]. Likewise, SOs may result in disappointment, confusion or increased uncertainty for patients. SOs may increase physician workload and might be perceived as signalling a patient's distrust, harming the doctor-patient relationship [17]. The cost-effectiveness of patient-initiated SOs has also been questioned; SOs may be costly if they involve additional consultations and diagnostic testing, or more expensive treatment recommendations [4,17,18]. In contrast, others have argued that SOs may reduce costs by preventing unnecessary treatment [4], which is a the rationale for insurer-mandated SOs.

A previous systematic review aimed to determine the clinical outcomes of patient-initiated SOs in general medical care, their satisfaction, characteristics and motivating factors for seeking SO [19]. The review reported that a surprising paucity of studies have examined the impact of patient-initiated SOs. Patients seeking a SO were mostly women with an average age of 54 years and a diagnosis of breast cancer. Generally, patients were satisfied with SOs, which were more often driven by

For peer review only

emotional factors than by concern about their own clinical outcomes. Common motivating factors for seeking a SO were having unresolved symptoms and treatment complications, dissatisfaction with their initial doctor, or seeking additional information. Overall, most patients perceived SOs to be valuable, either because of reassurance or the identification of an alternative [19]. Two other systematic reviews focus on SOs in oncology [13,20].

As new evidence has been accumulated since the last review, conducted in 2013 [19], we carried out an updated review. We designed a refined search strategy, as the previous review's search strategy consisted almost entirely of subject headings terms and referred to allied health-seeking behaviours such as doctor-shopping and medical nomadism. In three studies, data on patient-initiated SOs could not be separated from physician-initiated SOs.

We aimed to summarise evidence on (1) the characteristics and motivating factors of patients who initiate SOs; (2) the impact of patient-initiated SOs on diagnosis, treatment, prognosis and patient satisfaction; and (3) their cost-effectiveness.

Methods

Eligibility Criteria

A systematic review was performed following the Cochrane Handbook for Systematic Reviews of Interventions approach and using the Preferred Reporting Items for Systematic Reviews and Meta-Analyses (PRISMA) statement to report findings.[21,22] A second medical opinion was defined as a situation in which a patient, after getting a medical opinion from one doctor, obtained an opinion from another doctor regarding their diagnosis, treatment, or prognosis. Eligible studies were published in English-language scientific journals with patient-initiated SOs as the focus, which provided quantitative data on their impact on diagnosis, treatment, prognosis or patient satisfaction, described the characteristics or motivating factors of patients who initiated a SO, or analysed the cost-effectiveness of patient-initiated SOs. Studies that evaluated only physician-initiated referrals, mandatory or routine second reviews, SOs for legal reasons, online or over-the-phone SOs, or SOs in subspecialised domains such as dentistry and psychiatry, were excluded. Case studies, conference abstracts, comments, editorials, books and review articles were excluded.

Information Sources

A systematic literature search of Embase, Medline, PsycINFO and HMIC databases was performed. Search terms were keywords related to 'SO' and 'patient' (see appendix). Additional records were identified through hand searching (of reference lists of relevant papers?). No date restriction was applied. The searches were conducted in December 2019.

For peer review only

Study Selection

The records identified through database searching and hand searching were first de-duplicated. The titles and abstracts of the remaining records were then independently reviewed by two reviewers (AH and BH) to identify those meeting the inclusion criteria. 10% of the reviewed records were reviewed by another author (GG). Finally, the full text of eligible articles was independently reviewed by two reviewers (AH and BH). Eligibility differences throughout screening were reconciled through discussions.

Data Extraction and Quality Assessment

A data extraction form was developed and used to capture data elements. Study quality was assessed by AH, BH and GG using the National Institutes of Health (NIH) Quality Assessment Tool for Observational Cohort and Cross-Sectional Studies, with 14 questions being answered for each study [23].

Data Synthesis and Analysis

Evidence tables were constructed detailing the characteristics, medical specialties, results and quality of the studies. The outcome measures were then summarised.

Results

Database searching identified 4,004 records and hand searching identified one additional record (Figure 1). 1,252 records were excluded during deduplication, resulting in 2,753 unique records. Of these, 2,692 were excluded during title and abstract screening, leaving 61 potentially relevant articles. 30 articles were excluded during a full-text review; 31 articles were included in this review.

Study Characteristics

The 31 included articles described patients with cancer (n=17) and other medical domains (n=14) such as ophthalmology, orthopaedics and neurology (Table 1 and Table 2). Studies were performed in the U.S (n=10), Netherlands (n=7), Israel (n=5), Australia (n=2), Germany (n=3), Japan (n=2), Hong Kong and Scotland (both n=1). The 31 studies all used an observational design, either cross-sectional (n=28) or cohort (n=3). The sample size ranged between 36 to 208,366. Studies reported on patient characteristics (n=27), patient motivating factors (n=18), patient satisfaction (n=10) and clinical outcome agreement (n=17).

For peer review only

Risk of bias across studies

All studies used an observational design without control patients. All clearly defined their objective, study population, and exposure and outcome measures, and all consistently implemented across all study participants the inclusion and exclusion criteria for participation in the study, and the exposure and outcome measures. However, only 3 studies measured the exposures of interest prior to the outcomes being measured, and only 11 studies measured key potential confounding variables and adjusted them statistically for their impact on the relationship between exposures and outcomes. No studies blinded outcome assessors to the exposure status of participants. No studies evaluated the possibility of the SO having unintended consequences. The sample size of studies was often small, with 23 studies including fewer than 500 participants and 6 including under 100. Only 3 studies measured the exposures of interest prior to the outcomes being measured, and 11 studies statistically adjusted for potential confounders (Appendix 2).

Patient Characteristics associated with SO Seeking

More females than males had sought a SO: among patients with general medical concerns 52-61% of patients who sought a SO were female [12,24–31]; among cancer patients 77-87% of patients who sought a SO were female[32–34]. Conversely, two studies reported that more male cancer patients sought a SO than female [27,35].

Patients with a higher educational level sought SO more frequently[27,28,30,31,36–43].Most patients seeking a SO were middle aged. The mean age of cancer patients who sought a SO ranged from 49 to 59 years [44]. The mean age of patients with general medical concerns ranged from 44to 63 years [24–26,28,29,45,46].

Seeking a SO was common in non-religious patients vs. religious patients [42], in patients who were employed [37,39] and in patients with higher income and socioeconomic status [12,30,31,36,42]. SOs were more common among breast cancer patients who had a private insurance [37], and among men with localised prostate cancer with a private insurance [39]. Two studies reported on geographic residency, more common for those living in central areas [12] and for those closer to a SO centre [47]. Patients seeking a SO were more actively involved in decision-making processes [40], had a poorer relationship with their first doctor[47], were more anxious and believed they were in poor health [28].

Patient Motivating Factors

The most common reason reported for seeking a SO was to confirm or refute the suggested diagnosis or treatment or [26,32,34,35]; where patients disagreed with their doctor on diagnosis, 52% sought a

For peer review only - http://bmjopen.bmj.com/site/about/guidelines.xhtml

SO[44]. Undiagnosed complaints were reported by 85% of SO seekers [24], and 79% for a change in treatment [27]. For example, 59% of patients seeking a SO at a neurological clinic hoped for a different diagnosis or treatment than the first opinion [29]. Among orthopaedic patients, 38-40% questioned the first diagnosis or believed it was incorrect [47,48] and 18% sought reassurance about a recommended surgery [49]. 41% of ophthalmology patients sought a SO because their first doctor indicated that no treatment was possible, or that their prognosis was poor [25]. Patients often sought SOs where they disagreed with their doctor on proposed treatments (29% of drug-related disagreements, and 53% of other treatment disagreements) [44].

Patients often sought a SO to get more information related to diagnosis, treatment options and reassurance [50]. Some were seeking a sub-specialist's opinion [48], with the natural wish 'to be seen by the best doctor' [38]. Dissatisfaction with communication with the first doctor ranged from 19% [48] to 51% [47]. Some patients were encouraged by family members or friends to seek a SO [50], or were recommended a certain doctor by family or friends [47].

Patient Satisfaction

Patients were commonly very satisfied with the SO they received. The SO provided them with reassurance of their treatment or diagnosis, gaining comprehensible information about the treatment [35,50], with a compassionate approach addressing their needs [50] and obtaining answers to their concerns [34]. 84% of SO seekers among the general adult population in Israel were satisfied with the SO and 91% preferred the SO over the FO [48]. 95% of patients enrolled in a national SO program in the US were satisfied with the experience and 87% were more confident in their diagnosis or treatment [49]. In a survey conducted in Japan, most patients who obtained a SO reported they better understood their treatment options (93%), their illness (88%) and the risks of their treatment (82%) [27]. SO consultations in neurology received higher scores than the FO consultations across many aspects of satisfaction: patient involvement in the conversation and in decision-making, information and emotional support given [29]. However, during a 2 year follow-up study, overall satisfaction decreased to the same level as before the SO consultation [51]. 21 out of 37 parents of children with cancer in a paediatric haematology oncology department were satisfied with the second opinion they received [42].

Most patients in all studies were satisfied with their SO consultation. Patients reported feeling more knowledgeable and reassured about their diagnosis and treatment [34], and reported their trust in the attending physician was strengthened by getting a second opinion [35]. Some patients believed that the second doctor communicated better, answering concerns and providing more information (51%), listening more (39%) and being friendlier (41%) [34].

For peer review only

Clinical agreement between the first and SO

Substantial discrepancies between the first and SOs in diagnosis and suggested treatment were reported across the studies. Diagnosis was confirmed in 50% [27] to 57% [49] of cases, clarified in 17% and changed in 13% [27] to 15% [49]. Among women seeking a SO at a uterine fibroid treatment centre, 13.2% of previous diagnoses of uterine fibroids were unconfirmed by the SO [45]. In people who sought a SO for general medical concerns whilst enrolled in a national SO program, diagnosis was confirmed in 56.8% of cases, clarified in 17% and changed in 14.8% [49]. In patients seeking a SO at an eye hospital, there was 67.9% agreement with surgery recommendations between the FO and SO consultations [25]. Changes in both diagnosis and treatment were experienced by 11% [49] to 56% [29] of patients who sought a SO.

Among lung cancer patients, differences were found between the FO and the SO in 9% of diagnoses (17 patients) and in 13% of cancer stage classification (24 patients) and in 37% of therapeutic advice (70 patients). In total, there were 91 discrepancies between the FO and SO, of which 53 (58%) had a potential major impact on survival, morbidity and quality of life [52].

In surgical oncological cases where the second and first opinions could be directly compared, the advice was identical in 68%, there was a major discrepancy in 16% and a minor discrepancy in another 16% [33].

SO treatment recommended for surgical breast cancer deviated from the FO consultation in 20.3% of 54 cases [53]. 35% of 37 parents of children with haematological cancer were advised to change the treatment advised in the FO [42]. However, 56% of breast cancer patients didn't receive a recommendation for surgery either in their FO or SO consultation [43].

SOs received had a substantial impact of patient decision making. For 42% of cancer patients their SO consultation resulted in a change of treatment.[34] 68% of patients with general medical concerns mentioned they would change or partially change the treatment when the SO and FO differed [27].

Cost-effectiveness

No studies were found to report on the cost-effectiveness of patient-initiated SOs.

Discussion

Summary of findings

Women tended to seek SOs more than men. Most patients seeking a SO were middle aged, with a higher educational level. They tended to be employed, have a higher income and socioeconomic

For peer review only

status, and have private medical insurance. Patients seeking a SO sought to gain more information about their condition, gain reassurance about their diagnosis or treatment, were dissatisfied with their previous doctor or were encouraged by family members or friends to seek a SO.

Seeking SOs in many cases stemmed from dissatisfaction with the information and the communication with the first doctor, where patients felt they were not given the information or reassurance they sought. Most patients were satisfied with their SO consultation, felt more knowledgeable and reassured about their diagnosis and treatment, and reported having more confidence and trust in their second doctor. Patients believed that their second doctors communicated better, listened more and were friendlier.

A considerable proportion of SO consultations yielded a change in diagnosis or treatment, and these discrepancies had potentially major impact on patient outcomes in up to 58.2% of lung cancer cases. Despite the cost-effectiveness of routine and mandatory SO programs having been extensively studied [54–56], we found no studies reporting on the cost-effectiveness of patient-initiated SOs.

Strengths and Limitations

The review offers a broad overview on the topic of SOs and adds to the previous review in terms of breadth and up-to-dateness [19]. The previous review consisted almost entirely of subject headings search, and therefore likely to have missed relevant studies. Only eight of the thirteen studies contained data on patient initiated SOs. Two studies referred to doctor-shopping behaviour and to medical nomadism (where patients consult with multiple doctors for the same symptomatology during a certain period), which are different help-seeking behaviours than seeking a SO in terms of patient profile and motivation for seeking further advice. In three studies, data on patient-initiated SOs could not be separated from physician-initiated SOs. We aimed to overcome these limitations in this review.

Some limitations should be acknowledged. The main challenge in interpreting these findings is in the cohort of studies from different countries and different healthcare systems, where different insurance models are in place. For example, in some countries and under specific insurance schemes, access to SOs is covered by national and private insurers, whereas in other systems, SOs would be out-of-pocket. Differences in cultures and attitudes towards parallel consultations with different doctors may also affect the findings presented in studies in this review. Likewise, searching only for articles in the English-language means that we may have missed eligible articles in other languages.

Comparison with Previous Research

The review offers an updated and broader perspective on patient-initiated SOs. This review identified an additional 18 studies, 9 of which were published before the previous review [19]. Both reviews included only observational studies with an absence of data on control patients. Both reviews found no studies which evaluated the possibility of the SO having unintended consequences. Regarding the

For peer review only

characteristics of patients who had sought a SO, the previous review reported only that a large proportion of patients seeking a SO were women with an average age of 54 with a diagnosis of breast cancer. The education level of SO seekers ranged from those with less than a high school education to those with a university degree. This review referred to a broad range of factors pertaining to religious belief; employment, income and insurance; geographic residency; preference for involvement in decision-making; relationship with their first doctor; anxiety and beliefs they were in poor health.

We found similar motivating factors of patients compared to the previous review, with the vast majority of motivating factors for both cancer patients and patients with general medical concerns related to gaining more information about their condition, reassurance about their diagnosis or treatment, or dissatisfaction with their previous doctor. Both reviews found most patients in the studies to be satisfied with their SO consultation, however, a cohort study in this review reported that patient satisfaction dropped in the 2 years following the SO consultation to slightly below the satisfaction with the FO consultation. Both reviews found that SOs most typically confirm the original diagnosis or treatment, but that a considerable proportion of SOs yield a change. We also report that some medical specialties experience significantly more or fewer changes in diagnosis or treatment than average, and that changes in diagnosis and treatment have a more significant impact in cancer patients than in patients with general medical concerns. Two other systematic reviews focus specifically on SO in oncology [13,20]. We did not limit to specific medical specialties and so report evidence on SO in all medical domains.

Implications for practice

While SOs usually confirm the original diagnosis or treatment, a considerable proportion of SO consultations yield a change in treatment. Some medical specialties experienced significantly more changes in diagnosis or treatment, and changes in diagnosis and treatment had a more significant impact in cancer patients than in patients with general medical concerns. In specialities where there are often major discrepancies, there is a case to initiate a SO systematically or at least to make patients aware of the option of seeking a SO. Likewise, in cases where patients delay or avoid making a decision about a treatment course, SOs can help reassure and expedite the treatment. SO may benefit patients emotionally, even if they do not result in medical changes.

The fact that patients seeking a SO tended to be more educated patients, with higher income or socioeconomic status, having private insurance and living in central urban areas, raise concerns about inequalities and access to SOs among deprived groups and those living in rural areas, where access to specialists is limited.

While in many cases the SO confirms the FO, from the patient perspective, a change in their diagnosis or a treatment course may have a crucial impact on their lives, particularly in surgical oncology. From

For peer review only

the healthcare system or the insurer perspective, changes in diagnosis or treatment, even if they occur in only a portion of patients, may have substantial impact on patient outcomes, rehabilitation, costs and healthcare staff resources. For example, in the NHS in England, there is a legal requirement that every histopathology assessment should be by two pathologists, which is also built in the health system costing.

SOs stemming from unsatisfactory communication with the first doctor could be potentially avoided by improved doctor-patient communication, offering a detailed explanation and a listening approach. Rather than the SOs being sought confidentially, to not offend the first doctor, doctors should encourage a SO if they sense the patient is in doubt, and assist in referring the patient to a suitable consultant, and help to come to a mutual decision based on a discussion between the patient and both doctors. By negotiating a treatment that is acceptable to all parties, patients may be spared the confusion associated with discrepant opinions. By preparing patients for the various potential positive and negative outcomes of a SO, doctors can help them make an informed decision about pursuing the SO.

More people taking SO in national healthcare systems will put additional strain on the secondary care, but if unnecessary surgery is cancelled following a SO this will release resources, not to mention a long rehabilitation process which often follow surgery.

Future Research

Although our review suggests that patients generally believe SOs to be valuable, studies infrequently presented follow-up data on patient outcomes. It would also be useful to further explore the extent to which patients are referred back to their initial doctor, and to what extent SOs actually changed the course of treatment (rather than the mere fact that an additional opinion had been obtained). There is a distinct lack of studies on the cost-effectiveness of patient- initiated SOs, despite extensive literature on the cost-effectiveness of routine and mandatory SO programs. Long-term outcomes and potential unintended consequences of SOs must also be examined. Likewise, there is a lack of a uniform definition or objective measures of 'SO', which makes the comparison of findings across studies and health systems challenging. Development of uniform measures will be useful to uniformly compared findings across different countries and healthcare systems.

Conclusions

Seeking a second opinion was more common in women, middle age patients, more educated patients; and in people having a chronic condition, with higher income or socioeconomic status or living in central urban areas. Patients seeking a second opinion sought to gain more information or reassurance about their diagnosis or treatment. While many second opinions confirm the original diagnosis or treatment, discrepancies in opinions had a potential major impact on patient outcomes in up to 58.2%

For peer review only

of cases. No studies reporting on the cost-effectiveness of patient initiated second opinions. Research is needed to examine cost-effectiveness of second opinions and to identify patient groups that are likely to benefit from a second opinion. In the context of rising pressure on primary and secondary care services, it is important to set up clear mechanisms for patients seeking second opinions in both public and private systems.

For peer review only

Contributorship statement

GG, LS and BH were involved with conception and design, conducted the data analysis, and drafted the manuscript. AH and HQY were involved in designing and conducting the literature searches, screening, data extraction and synthesis, and revised various versions of the manuscript. ND, JP, SR and AM were involved in conception and design, interpretation of the findings, provided clinical perspectives, and revised various versions of the manuscript.

Data sharing statement

All data are publicly available.

Funding

This report is independent research supported by the National Institute for Health Research Applied Research Collaboration Northwest London. The views expressed in this publication are those of the authors and not necessarily those of the National Institute for Health Research or the Department of Health and Social Care.

Ethics

The study did not require an Ethics approval.

Competing Interest

None declared.

Patient and Public Involvement statement

Patients or the public were not involved in the design, or conduct, or reporting, or dissemination plans of our research.

For peer review only

References

1 Tam KF, Cheng DKL, Ng TY, *et al.* The behaviors of seeking a second opinion from other health-care professionals and the utilization of complementary and alternative medicine in gynecologic cancer patients. *Support Care Cancer Off J Multinatl Assoc Support Care Cancer* 2005;**13**:679–84. doi:10.1007/s00520-005-0841-4

2 Rosenberg SN, Gorman SA, Snitzer S, *et al.* Patients' reactions and physician-patient communication in a mandatory surgical second-opinion program. *Med Care* 1989;**27**:466–77.

3 Odeyemi IA, Nixon J. The role and uptake of private health insurance in different health care systems: are there lessons for developing countries? *Clin Outcomes Res CEOR* 2013;**5**:109–18. doi:10.2147/CEOR.S40386

4 Moumjid N, Gafni A, Bremond A, *et al.* Seeking a second opinion: do patients need a second opinion when practice guidelines exist? *Health Policy Amst Neth* 2007;**80**:43–50. doi:10.1016/j.healthpol.2006.02.009

5 Davidov T, Trooskin SZ, Shanker B-A, *et al.* Routine second-opinion cytopathology review of thyroid fine needle aspiration biopsies reduces diagnostic thyroidectomy. *Surgery* 2010;**148**:1294–301. doi:10.1016/j.surg.2010.09.029

6 Allen TC. Second Opinions: Pathologists' Preventive Medicine. *Arch Pathol Lab Med* 2013;**137**:310–1. doi:10.5858/arpa.2012-0512-ED

7 Raab SS, Grzybicki DM, Janosky JE, *et al.* Clinical impact and frequency of anatomic pathology errors in cancer diagnoses. *Cancer* 2005;**104**:2205–13. doi:10.1002/cncr.21431

8 Tomaszewski JE, Bear HD, Connally JA, *et al.* Consensus Conference on Second Opinions in Diagnostic Anatomic Pathology. *Am J Clin Pathol* 2000;**114**:329–35. doi:10.1093/ajcp/114.3.329

9 Moumjid N, Gafni A, Bremond A, *et al.* Seeking a second opinion: do patients need a second opinion when practice guidelines exist? *Health Policy Amst Neth* 2007;**80**:43–50.

10 Sutherland LR, Verhoef MJ. Why do patients seek a second opinion or alternative medicine? *J Clin Gastroenterol* 1994;**19**:194–7.

11 Wagner T, Wagner L. Who gets second opinions? *Health Aff Proj Hope* 1999;**18**:137–45.

For peer review only

12 Shmueli L, Shmueli E, Pliskin JS, *et al.* Second Medical Opinion: Utilization Rates and Characteristics of Seekers in a General Population. *Med Care* 2016;**54**:921–8. doi:10.1097/MLR.0000000000000567

13 Hillen MA, Medendorp NM, Daams JG, *et al.* Patient-Driven Second Opinions in Oncology: A Systematic Review. *The Oncologist* 2017;**22**:1197–211. doi:10.1634/theoncologist.2016-0429

14 Berner et al. ES. Overconfidence as a Cause of Diagnostic Error in Medicine. *Am J Med* 2008;**121**:S2–23. doi:10.1016/j.amjmed.2008.01.001

15 Graber ML. The incidence of diagnostic error in medicine. *BMJ Qual Saf* 2013;**22**:ii21–7. doi:10.1136/bmjqs-2012-001615

16 Singh H, Giardina TD, Meyer AND, *et al.* Types and Origins of Diagnostic Errors in Primary Care Settings. *JAMA Intern Med* 2013;**173**:418–25. doi:10.1001/jamainternmed.2013.2777

17 Axon A, Hassan M, Niv Y, *et al.* Ethical and legal implications in seeking and providing a second medical opinion. *Dig Dis* 2008;**26**:11–7.

18 Mustafa et al. MK. What is the value of patient-sought second opinions? *Eur J Intern Med* 2002;**13**:445. doi:10.1016/S0953-6205(02)00138-3

19 Payne VL, Singh H, Meyer AND, *et al.* Patient-initiated second opinions: systematic review of characteristics and impact on diagnosis, treatment, and satisfaction. *Mayo Clin Proc* 2014;**89**:687–96. doi:10.1016/j.mayocp.2014.02.015

20 Ruetters D, Keinki C, Schroth S, *et al.* Is there evidence for a better health care for cancer patients after a second opinion? A systematic review. *J Cancer Res Clin Oncol* 2016;**142**:1521–8. doi:10.1007/s00432-015-2099-7

21 Cochrane Handbook for Systematic Reviews of Interventions. 2011.https://handbook-5-1.cochrane.org/ (accessed 25 Sep 2019).

22 Moher D, Liberati A, Tetzlaff J, *et al.* Preferred Reporting Items for Systematic Reviews and Meta-Analyses: The PRISMA Statement. *PLoS Med* 2009;**6**:e1000097. doi:10.1371/journal.pmed.1000097

23 Study Quality Assessment Tools | National Heart, Lung, and Blood Institute (NHLBI). Natl. Inst. Health. https://www.nhlbi.nih.gov/health-topics/study-quality-assessment-tools (accessed 25 Sep 2019).

For peer review only

24 Mustafa MK, Bijl M, Gans ROB. What is the value of patient-sought second opinions? *Eur J Intern Med* 2002;**13**:445–7. doi:10.1016/S0953-6205(02)00138-3

25 Benson WE, Regillo CD, Vander JF, *et al.* PATIENT–INITIATED SECOND MEDICAL OPINIONS: Their Necessity and Economic Cost. *RETINA* 2001;**21**:633.

26 Gologorsky D, Greenstein S. Retrospective analysis of patients self-referred to comprehensive ophthalmology seeking second opinions. *Clin Ophthalmol* 2013;:1099. doi:10.2147/OPTH.S46448

27 Okamoto S, Kawahara K, Okawa A, *et al.* Values and risks of second opinion in Japan's universal health-care system. *Health Expect* 2015;**18**:826–38. doi:10.1111/hex.12055

28 Sato T, Takeichi M, Hara T, *et al.* Second opinion behaviour among Japanese primary care patients. *Br J Gen Pract J R Coll Gen Pract* 1999;**49**:546–50.

29 Wijers D, Wieske L, Vergouwen MDI, *et al.* Patient satisfaction in neurological second opinions and tertiary referrals. *J Neurol* 2010;**257**:1869–74. doi:10.1007/s00415-010-5625-1

30 Cecon N, Hillen MA, Pfaff H, *et al.* Why do newly diagnosed breast cancer patients seek a second opinion? – Second opinion seeking and its association with the physician-patient relationship. *Patient Educ Couns* 2019;**102**:998–1004. doi:10.1016/j.pec.2018.12.017

31 Shmueli L, Davidovitch N, Pliskin JS, *et al.* Reasons, perceived outcomes and characteristics of second-opinion seekers: are there differences in private vs. public settings? *BMC Health Serv Res* 2019;**19**:238. doi:10.1186/s12913-019-4067-4

32 Mellink WA, Dulmen AM, Wiggers T, *et al.* Cancer patients seeking a second surgical opinion: results of a study on motives, needs, and expectations. *J Clin Oncol Off J Am Soc Clin Oncol* 2003;**21**:1492–7.

33 Mellink WAM, Henzen-Logmans SC, Bongaerts AHH, *et al.* Discrepancy between second and first opinion in surgical oncological patients. *Eur J Surg Oncol EJSO* 2006;**32**:108–12. doi:10.1016/j.ejso.2005.08.007

34 Tattersall MHN, Dear RF, Jansen J, *et al.* Second opinions in oncology: the experiences of patients attending the Sydney Cancer Centre. *Med J Aust* 2009;**191**:209–12.

35 Fuchs T, Hanaya H, Seilacher E, *et al.* Information Deficits and Second Opinion Seeking – A Survey on Cancer Patients. *Cancer Invest* 2017;**35**:62–9. doi:10.1080/07357907.2016.1242012

For peer review only

36 Tam KF, Cheng DK, Ng TY, *et al.* The behaviors of seeking a second opinion from other health-care professionals and the utilization of complementary and alternative medicine in gynecologic cancer patients. *Support Care Cancer Off J Multinatl Assoc Support Care Cancer* 2005;**13**:679–84.

37 Katz SJ, Janz NK, Abrahamse P, *et al.* Patient Reactions to Surgeon Recommendations About Contralateral Prophylactic Mastectomy for Treatment of Breast Cancer. *JAMA Surg* 2017;**152**:658. doi:10.1001/jamasurg.2017.0458

38 Radhakrishnan A, Grande D, Mitra N, *et al.* Second opinions from urologists for prostate cancer: Who gets them, why, and their link to treatment: Second Opinions From Urologists for Prostate Cancer. *Cancer* 2017;**123**:1027–34. doi:10.1002/cncr.30412

39 Ramsey SD, Zeliadt SB, Fedorenko CR, *et al.* Patient preferences and urologist recommendations among local-stage prostate cancer patients who present for initial consultation and second opinions. *World J Urol* 2011;**29**:3–9. doi:10.1007/s00345-010-0602-y

40 Groß SE, Hillen MA, Pfaff H, *et al.* Second opinion in medical encounters − A study among breast cancer patients. *Patient Educ Couns* 2017;**100**:1990–5. doi:10.1016/j.pec.2017.05.038

41 Kurian AW, Friese CR, Bondarenko I, *et al.* Second Opinions From Medical Oncologists for Early-Stage Breast Cancer: Prevalence, Correlates, and Consequences. *JAMA Oncol* 2017;**3**:391. doi:10.1001/jamaoncol.2016.5652

42 Mordechai O, Tamir S, Weyl-Ben-Arush M. Seeking a Second Opinion in Pediatric Oncology. *Pediatr Hematol Oncol* 2015;**32**:284–9. doi:10.3109/08880018.2014.987938

43 Morrow M, Jagsi R, Alderman AK, *et al.* Surgeon recommendations and receipt of mastectomy for treatment of breast cancer. *JAMA J Am Med Assoc* 2009;**302**:1551–6.

44 Annandale E, Hunt K. Accounts of disagreements with doctors. *Soc Sci Med* 1998;**46**:119–29. doi:10.1016/S0277-9536(97)00149-4

45 Tan N, McClure TD, Tarnay C, *et al.* Women seeking second opinion for symptomatic uterine leiomyoma: role of comprehensive fibroid center. *J Ther Ultrasound* 2014;**2**:3. doi:10.1186/2050-5736-2-3

46 Shmueli L, Shmueli E, Pliskin JS, *et al.* Second opinion utilization by healthcare insurance type in a mixed private-public healthcare system: a population-based study. *BMJ Open* 2019;**9**:e025673. doi:10.1136/bmjopen-2018-025673

For peer review only

47 van Dalen I, Groothoff J, Stewart R, *et al.* Motives for seeking a second opinion in orthopaedic surgery. *J Health Serv Res Policy* 2001;**6**:195–201.

48 Shmueli L, Davidovitch N, Pliskin JS, *et al.* Seeking a second medical opinion: composition, reasons and perceived outcomes in Israel. *Isr J Health Policy Res* 2017;**6**:67. doi:10.1186/s13584-017-0191-y

49 Meyer AND, Singh H, Graber ML. Evaluation of Outcomes From a National Patient-initiated Second-opinion Program. *Am J Med* 2015;**128**:1138.e25-1138.e33. doi:10.1016/j.amjmed.2015.04.020

50 Philip J, Gold M, Schwarz M, *et al.* Second medical opinions: the views of oncology patients and their physicians. *Support Care Cancer* 2010;**18**:1199–205. doi:10.1007/s00520-009-0742-z

51 Wieske L, Richard E, Wijers D, *et al.* Long-term satisfaction after neurological second opinions and tertiary referrals. *Eur J Neurol* 2011;**18**:1310–6. doi:10.1111/j.1468-1331.2011.03394.x

52 Schook RM, Ter Avest MJ, Van Setten CH, *et al.* Lung Cancer Patients Benefit from Second Opinions by Improvement of Diagnosis and Therapy. *Cancer Clin Oncol* 2014;**3**:p43. doi:10.5539/cco.v3n1p43

53 Clauson J, Hsieh YC, Acharya S, *et al.* Results of the Lynn Sage Second-Opinion Program for local therapy in patients with breast carcinoma. Changes in management and determinants of where care is delivered. *Cancer* 2002;**94**:889–94.

54 I. Epstein et al. J, Fred. Clinical and Cost Impact of Second-opinion Pathology. *Am J Surg Pathol* 1996;**20**:851–7. doi:10.1097/00000478-199607000-00008

55 Lindsey PA, Newhouse JP. The Cost and Value of Second Surgical Opinion Programs: A Critical Review of the Literature. *J Health Polit Policy Law* 1990;**15**:543–70. doi:10.1215/03616878-15-3-543

56 Torok CM, Lee C, Nagy P, *et al.* Neuroradiology Second Opinion Consultation Service: Assessment of Duplicative Imaging. *Am J Roentgenol* 2013;**201**:1096–100. doi:10.2214/AJR.12.9429

For peer review only

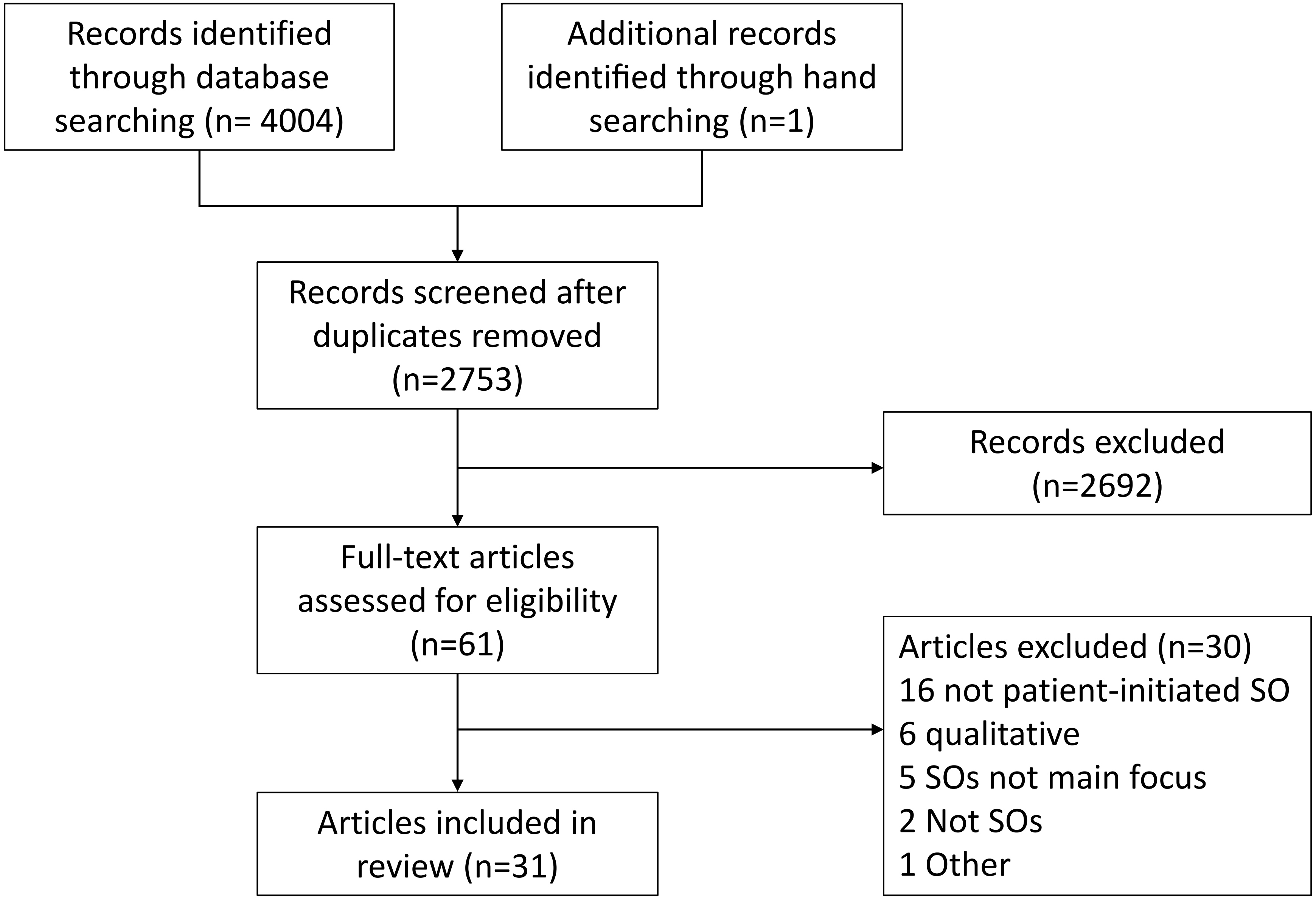
Records identified through database searching (n= 4004)
Additional records identified through hand searching (n=1)
Records screened after duplicates removed (n=2753)
Records excluded (n=2692)
Full-text articles assessed for eligibility (n=61)
Articles excluded (n=30)
16 not patient-initiated SO
6 qualitative
5 SOs not main focus
2 Not SOs
1 Other
Articles included in review (n=31)

Appendix 1

Search strategy

((second adj2 opinion*) OR (second adj2 consult*)) AND patient*

Appendix 2

Study quality assessment

Study quality was assessed using the NIH Quality Assessment Tool for Observational Cohort and Cross-Sectional Studies.[34] The following 14 questions were answered for each study:

1. Was the research question or objective in this paper clearly stated?

2. Was the study population clearly specified and defined?

3. Was the participation rate of eligible persons at least 50%?

4. Were all the subjects selected or recruited from the same or similar populations (including the same time period)? Were inclusion and exclusion criteria for being in the

study prespecified and applied uniformly to all participants?

5. Was a sample size justification, power description, or variance and effect estimates provided?

6. For the analyses in this paper, were the exposure(s) of interest measured prior to the outcome(s) being measured?

7. Was the timeframe sufficient so that one could reasonably expect to see an association between exposure and outcome if it existed?

8. For exposures that can vary in amount or level, did the study examine different levels of the exposure as related to the outcome (e.g., categories of exposure, or exposure measured as continuous variable)?

9. Were the exposure measures (independent variables) clearly defined, valid, reliable, and implemented consistently across all study participants?

10. Was the exposure(s) assessed more than once over time?

11. Were the outcome measures (dependent variables) clearly defined, valid, reliable, and implemented consistently across all study participants?

12. Were the outcome assessors blinded to the exposure status of participants?

For peer review only

13. Was loss to follow-up after baseline 20% or less?

14. Were key potential confounding variables measured and adjusted statistically for their impact on the relationship between exposure(s) and outcome(s)?

For peer review only

Table 1: Study characteristics

Study	Medical specialty	Location	Study design	No. of participants	Characteristics	Motivation	Satisfaction	Diagnosis	Treatment	Prognosis
Clauson, 2002[53]	Breast cancer	United States	Cross-sectional	231	X				X	
Fuchs, 2017 [35]	Cancer	Germany	Cross-sectional	36	X	X	X		X	
Groß, 2017[40]	Breast cancer	Germany	Cross-sectional	2846	X	X				
Katz, 2017[37]	Breast cancer	United States	Cross-sectional	304	X					
Kurian, 2017[41]	Breast cancer	United States	Cross-sectional	168	X					
Mellink, 2003[32]	Cancer	Netherlands	Cross-sectional	212	X	X				
Mellink, 2006[33]	Cancer	Netherlands	Cohort	403	X			X	X	X

For peer review only

Study	Medical specialty	Location	Study design	No. of participants	Characteristics	Motivation	Satisfaction	Diagnosis	Treatment	Prognosis
Mordechai, 2015[42]	Haematological cancer	Israel	Cross-sectional	37	X		X		X	
Morrow, 2009[43]	Breast cancer	United States	Cross-sectional	378	X				X	
Philip, 2010[50]	Cancer	Australia	Cross-sectional	17/65*	X	X	X			
Radhakrishnan, 2017[38]	Prostate cancer	United States	Cross-sectional	950	X	X				
Ramsey, 2011[39]	Prostate cancer	United States	Cohort	143/25*	X				X	
Schook, 2014[52]	Lung cancer	Netherlands	Cross-sectional	184	X			X	X	
Tam, 2005[36]	Gynaecologic cancer	Hong Kong	Cross-sectional	80	X	X				
Tattersall, 2009[34]	Cancer	Australia	Cross-sectional	77	X	X	X		X	

For peer review only

Study	Medical specialty	Location	Study design	No. of participants	Characteristics	Motivation	Satisfaction	Diagnosis	Treatment	Prognosis
Annandale, 1998[44]	Gynaecology, respiratory, cardiovascular, other	Scotland	Cross-sectional	136		X				
Benson, 2001[25]	Ophthalmology	United States	Cross-sectional	100	X	X			X	
Gologorsky, 2013[26]	Ophthalmology	United States	Cross-sectional	174	X	X				
Meyer, 2015[49]	Orthopaedics, oncology, haematology, other	United States	Cross-sectional	6791		X	X	X	X	
Mustafa, 2002[24]	Fatigue, abdominal pain, chest pain, other	Netherlands	Cross-sectional	201	X	X		X	X	

For peer review only

Study	Medical specialty	Location	Study design	No. of participants	Characteristics	Motivation	Satisfaction	Diagnosis	Treatment	Prognosis
Okamoto, 2013[27]	Cancer, neurology, orthopaedics, other	Japan	Cross-sectional	149	X	X	X	X	X	
Sato, 1999[28]	Obstetrics, gynaecology, gastroenterology, other	Japan	Cross-sectional	420	X					
Shmueli, 2016[12]	Orthopaedics, ophthalmology, dermatology, other	Israel	Cross-sectional	208,366	X					
Shmueli, 2017[48]	Orthopaedics, ophthalmology, dermatology, other	Israel	Cross-sectional	344		X	X	X	X	

For peer review only

Study	Medical specialty	Location	Study design	No. of participants	Characteristics	Motivation	Satisfaction	Diagnosis	Treatment	Prognosis
Tan, 2014[45]	Gynaecology	United States	Cross-sectional	205	X			X	X	
Van Dalen, 2001[47]	Orthopaedics	Netherlands	Cross-sectional	401-411/349*	X	X				
Wieske, 2011[51]	Neurology	Netherlands	Cohort	76			X			
Wijers, 2010 [29]	Neurology	Netherlands	Cross-sectional	183	X	X	X	X	X	
Shmueli. 2019 [46]	Orthopaedics, ophthalmology, dermatology, other	Israel	Cross-sectional	143,371	X					
Cecon. 2019[30]	Breast cancer	Germany	Cross-sectional	419	X	X		X	X	

For peer review only

Study	Medical specialty	Location	Study design	No. of participants	Characteristics	Motivation	Satisfaction	Diagnosis	Treatment	Prognosis
Shmueli. 2019a[31]	Orthopaedics, ophthalmology, dermatology, other	Israel	Cross-sectional	339	X	X	X	X	X	

* indicates first doctors of patients who also participated in the study

For peer review only

Table 2: Quality assessment of studies

Study	1	2	3	4	5	6	7	8	9	10	11	12	13	14
Cecon, 2019 [30]	Y	Y	Y	Y	N	N	NA	NA	Y	NA	Y	N	NA	N
Clauson, 2002 [53]	Y	Y	NR	Y	Y	N	NA	NA	Y	NA	Y	N	NA	N
Fuchs, 2017 [35]	Y	Y	N	Y	N	N	NA	NA	Y	NA	Y	N	NA	N
Groß, 2017 [40]	Y	Y	Y	Y	Y	N	NA	NA	Y	NA	Y	N	NA	Y
Katz, 2017 [37]	Y	Y	Y	Y	N	N	NA	NA	Y	NA	Y	N	NA	Y
Kurian, 2017 [41]	Y	Y	Y	Y	Y	N	NA	NA	Y	NA	Y	N	NA	Y
Mellink, 2003 [32]	Y	Y	Y	Y	Y	N	NA	NA	Y	NA	Y	N	NA	N
Mellink, 2006 [33]	Y	Y	Y	Y	Y	Y	NA	NA	Y	NA	Y	N	Y	N
Mordechai, 2015 [42]	Y	Y	Y	Y	Y	N	NA	NA	Y	NA	Y	N	NA	N
Morrow, 2009 [43]	Y	Y	Y	Y	Y	N	NA	NA	Y	NA	Y	N	NA	N
Philip, 2010 [50]	Y	Y	Y	Y	N	N	NA	NA	Y	NA	Y	N	NA	N
Radhakrishnan, 2017 [38]	Y	Y	Y	Y	Y	N	NA	NA	Y	NA	Y	N	NA	Y
Ramsey, 2011 [39]	Y	Y	Y	Y	Y	Y	NA	NA	Y	NA	Y	N	Y	Y
Schook, 2014 [52]	Y	Y	Y	Y	Y	N	NA	NA	Y	NA	Y	N	NA	N
Tam, 2005 [36]	Y	Y	Y	Y	Y	N	NA	NA	Y	NA	Y	N	NA	Y

Study	1	2	3	4	5	6	7	8	9	10	11	12	13	14
Tattersall, 2009 [34]	Y	Y	Y	Y	N	N	NA	NA	Y	NA	Y	N	NA	N
Annandale, 1998 [44]	Y	Y	NR	Y	N	N	NA	NA	Y	NA	Y	N	NA	N
Benson, 2001 [25]	Y	Y	NR	Y	N	N	NA	NA	Y	NA	Y	N	NA	N
Gologorsky, 2013 [26]	Y	Y	Y	Y	N	N	NA	NA	Y	NA	Y	N	NA	N
Meyer, 2015 [49]	Y	Y	Y	Y	Y	N	NA	NA	Y	NA	Y	N	NA	N
Mustafa, 2002 [24]	Y	Y	Y	Y	N	N	NA	NA	Y	NA	Y	N	NA	N
Okamoto, 2013 [27]	Y	Y	Y	Y	N	N	NA	NA	Y	NA	Y	N	NA	N
Sato, 1999 [28]	Y	Y	Y	Y	Y	N	NA	NA	Y	NA	Y	N	NA	Y
Shmueli, 2016 [12]	Y	Y	Y	Y	Y	N	NA	NA	Y	NA	Y	N	NA	Y
Shmueli, 2017 [48]	Y	Y	Y	Y	Y	N	NA	NA	Y	NA	Y	N	NA	N
Shmueli, 2019 [46]	Y	Y	Y	Y	N	N	NA	NA	Y	NA	Y	N	NA	Y
Shmueli, 2019a [31]	Y	Y	Y	Y	Y	N	NA	NA	Y	NA	Y	N	NA	N
Tan, 2014 [45]	Y	Y	Y	Y	Y	N	NA	NA	Y	NA	Y	N	NA	N
Van Dalen, 2001 [47]	Y	Y	Y	Y	Y	N	NA	NA	Y	NA	Y	N	NA	Y
Wieske, 2011 [51]	Y	Y	N	Y	Y	Y	NA	NA	Y	NA	Y	N	N	Y
Wijers, 2010 [29]	Y	Y	Y	Y	Y	N	NA	NA	Y	NA	Y	N	NA	Y

Y, Yes; N, No; NR, Not Reported; NA, Not Applicable

For peer review only

PRISMA 2009 Checklist

Section/topic	#	Checklist item	Reported on page #
**TITLE**			
Title	1	Identify the report as a systematic review, meta-analysis, or both.	1
**ABSTRACT**			
Structured summary	2	Provide a structured summary including, as applicable: background; objectives; data sources; study eligibility criteria, participants, and interventions; study appraisal and synthesis methods; results; limitations; conclusions and implications of key findings; systematic review registration number.	2
**INTRODUCTION**			
Rationale	3	Describe the rationale for the review in the context of what is already known.	3
Objectives	4	Provide an explicit statement of questions being addressed with reference to participants, interventions, comparisons, outcomes, and study design (PICOS).	3
**METHODS**			
Protocol and registration	5	Indicate if a review protocol exists, if and where it can be accessed (e.g., Web address), and, if available, provide registration information including registration number.	N/A
Eligibility criteria	6	Specify study characteristics (e.g., PICOS, length of follow-up) and report characteristics (e.g., years considered, language, publication status) used as criteria for eligibility, giving rationale.	5
Information sources	7	Describe all information sources (e.g., databases with dates of coverage, contact with study authors to identify additional studies) in the search and date last searched.	5
Search	8	Present full electronic search strategy for at least one database, including any limits used, such that it could be repeated.	5
Study selection	9	State the process for selecting studies (i.e., screening, eligibility, included in systematic review, and, if applicable, included in the meta-analysis).	6
Data collection process	10	Describe method of data extraction from reports (e.g., piloted forms, independently, in duplicate) and any processes for obtaining and confirming data from investigators.	6
Data items	11	List and define all variables for which data were sought (e.g., PICOS, funding sources) and any assumptions and simplifications made.	6
Risk of bias in individual studies	12	Describe methods used for assessing risk of bias of individual studies (including specification of whether this was done at the study or outcome level), and how this information is to be used in any data synthesis.	6
Summary measures	13	State the principal summary measures (e.g., risk ratio, difference in means).	6
Synthesis of results	14	Describe the methods of handling data and combining results of studies, if done, including measures of consistency (e.g., I^2) for each meta-analysis.	6

For peer review only - http://bmjopen.bmj.com/site/about/guidelines.xhtml

PRISMA 2009 Checklist

Section/topic	#	Checklist item	Reported on page #
Risk of bias across studies	15	Specify any assessment of risk of bias that may affect the cumulative evidence (e.g., publication bias, selective reporting within studies).	6
Additional analyses	16	Describe methods of additional analyses (e.g., sensitivity or subgroup analyses, meta-regression), if done, indicating which were pre-specified.	6
**RESULTS**			
Study selection	17	Give numbers of studies screened, assessed for eligibility, and included in the review, with reasons for exclusions at each stage, ideally with a flow diagram.	6
Study characteristics	18	For each study, present characteristics for which data were extracted (e.g., study size, PICOS, follow-up period) and provide the citations.	6
Risk of bias within studies	19	Present data on risk of bias of each study and, if available, any outcome level assessment (see item 12).	7
Results of individual studies	20	For all outcomes considered (benefits or harms), present, for each study: (a) simple summary data for each intervention group (b) effect estimates and confidence intervals, ideally with a forest plot.	7-9
Synthesis of results	21	Present results of each meta-analysis done, including confidence intervals and measures of consistency.	7-9
Risk of bias across studies	22	Present results of any assessment of risk of bias across studies (see Item 15).	29
Additional analysis	23	Give results of additional analyses, if done (e.g., sensitivity or subgroup analyses, meta-regression [see Item 16]).	n/a
**DISCUSSION**			
Summary of evidence	24	Summarize the main findings including the strength of evidence for each main outcome; consider their relevance to key groups (e.g., healthcare providers, users, and policy makers).	9
Limitations	25	Discuss limitations at study and outcome level (e.g., risk of bias), and at review-level (e.g., incomplete retrieval of identified research, reporting bias).	10
Conclusions	26	Provide a general interpretation of the results in the context of other evidence, and implications for future research.	12
**FUNDING**			
Funding	27	Describe sources of funding for the systematic review and other support (e.g., supply of data); role of funders for the systematic review.	19

From: Moher D, Liberati A, Tetzlaff J, Altman DG, The PRISMA Group (2009). Preferred Reporting Items for Systematic Reviews and Meta-Analyses: The PRISMA Statement. PLoS Med 6(6): e1000097. doi:10.1371/journal.pmed1000097

For more information, visit: **www.prisma-statement.org**.

For peer review only - http://bmjopen.bmj.com/site/about/guidelines.xhtml

BMJ Open

Patient-initiated second medical consultations: patient characteristics and motivating factors, impact on care and satisfaction: A systematic review

Journal:	*BMJ Open*
Manuscript ID	bmjopen-2020-044033.R1
Article Type:	Original research
Date Submitted by the Author:	19-May-2021
Complete List of Authors:	Greenfield, Geva; Imperial College London Department of Life Sciences, Department of Primary Care and Public Health, School of Public Health<br>Shmueli, Liora; Ben-Gurion University of the Negev, Department of Health Systems Management; Tel Aviv University - Recanati Graduate School of Business Administration ,<br>Harvey, Amy; Imperial College London School of Public Health<br>Quezada-Yamamoto, Harumi; Imperial College London, Department of Primary Care and Public Health<br>Davidovitch, Nadav; Ben-Gurion University of the Negev, Department of Health Systems Management<br>Pliskin, Joseph ; Ben-Gurion University of the Negev<br>Rawaf, Salman; Imperial College London, Primary care and Public Health<br>Majeed, Azeem; Imperial College, Primary Care<br>Hayhoe, Benedict; Imperial College London School of Public Health
**Primary Subject Heading**:	Health services research
Secondary Subject Heading:	Health policy
Keywords:	GENERAL MEDICINE (see Internal Medicine), HEALTH SERVICES ADMINISTRATION & MANAGEMENT, Quality in health care < HEALTH SERVICES ADMINISTRATION & MANAGEMENT

SCHOLARONE™ Manuscripts

For peer review only - http://bmjopen.bmj.com/site/about/guidelines.xhtml

I, the Submitting Author has the right to grant and does grant on behalf of all authors of the Work (as defined in the below author licence), an exclusive licence and/or a non-exclusive licence for contributions from authors who are: i) UK Crown employees; ii) where BMJ has agreed a CC-BY licence shall apply, and/or iii) in accordance with the terms applicable for US Federal Government officers or employees acting as part of their official duties; on a worldwide, perpetual, irrevocable, royalty-free basis to BMJ Publishing Group Ltd ("BMJ") its licensees and where the relevant Journal is co-owned by BMJ to the co-owners of the Journal, to publish the Work in this journal and any other BMJ products and to exploit all rights, as set out in our licence.

The Submitting Author accepts and understands that any supply made under these terms is made by BMJ to the Submitting Author unless you are acting as an employee on behalf of your employer or a postgraduate student of an affiliated institution which is paying any applicable article publishing charge ("APC") for Open Access articles. Where the Submitting Author wishes to make the Work available on an Open Access basis (and intends to pay the relevant APC), the terms of reuse of such Open Access shall be governed by a Creative Commons licence – details of these licences and which Creative Commons licence will apply to this Work are set out in our licence referred to above.

Other than as permitted in any relevant BMJ Author's Self Archiving Policies, I confirm this Work has not been accepted for publication elsewhere, is not being considered for publication elsewhere and does not duplicate material already published. I confirm all authors consent to publication of this Work and authorise the granting of this licence.

For peer review only

Patient-initiated second medical consultations: patient characteristics and motivating factors, impact on care and satisfaction: A systematic review

Geva Greenfield[1], Liora Shmueli[2], Amy Harvey[1], Harumi Quezada-Yamamoto[1], Nadav Davidovitch[3], Joseph Pliskin[3], Salman Rawaf[1], Azeem Majeed[1], Benedict Hayhoe[1]

[1] Department of Primary Care and Public Health, School of Public Health, Imperial College London, UK

[2] Department of Management, Bar Ilan University, Ramat Gan, Israel

[3] Department of Health Systems Management and Department of Industrial Engineering & Management, Ben-Gurion University of the Negev, Beer-Sheva, Israel

* **Corresponding author**: Geva Greenfield, Department of Primary Care and Public Health, School of Public Health, Imperial College London, 314 The Reynolds Building, St Dunstan's Road, London W6 8RP, g.greenfield@ic.ac.uk

The manuscript includes 2,995 words, 56 references, 1 figure, and 2 tables. The abstract includes 291 words.

For peer review only

Abstract

Objectives: To review the characteristics and motivations of patients seeking second opinions, and the impact of such opinions on patient management, satisfaction, and cost-effectiveness.

Data sources: Embase, Medline, PsycINFO and HMIC databases.

Study design: A systematic literature search was performed for terms related to second opinion and patient characteristics. Study quality was assessed using the National Institutes of Health (NIH) Quality Assessment Tool for Observational Cohort and Cross-Sectional Studies.

Data collection / Extraction methods: We included articles focused on patient-initiated second opinions, which provided quantitative data on their impact on diagnosis, treatment, prognosis or patient satisfaction, described the characteristics or motivating factors of patients who initiated a second opinion, or the cost-effectiveness of patient-initiated second opinions.

Principal findings: Thirty-one articles were included in the review. 27 studies considered patient characteristics, 18 patient motivating factors, 10 patient satisfaction, and 17 clinical agreement between the first and second opinion. Seeking a second opinion was more common in women, middle age patients, more educated patients; and in people having a chronic condition, with higher income or socioeconomic status or living in central urban areas. Patients seeking a second opinion sought to gain more information or reassurance about their diagnosis or treatment. While many second opinions confirm the original diagnosis or treatment, discrepancies in opinions had a potential major impact on patient outcomes in up to 58% of cases. No studies reporting on the cost-effectiveness of patient initiated second opinions.

Conclusions: Seeking a second opinion was more common in women, middle-age patients, and more educated patients, and in people having a chronic condition, with higher income or socioeconomic status or living in central urban areas. Patients seeking a second opinion sought to gain more information or reassurance about their diagnosis or treatment.

Keywords: Second opinion, decision making, diagnostic discrepancies, cost-effectiveness, help-seeking behaviours.

For peer review only

Strengths and limitations of this study

- This review provides an up-to-date summary of the scientific literature on patient-initiated second medical consultations and adds to a previous review in its breadth
- The main challenge was in interpretation of findings from different countries with different healthcare systems and different health insurance models
- Searching for articles in the English-language only means that eligible articles in other languages may have been missed.

For peer review only

Background

A second medical opinion (SO) is a medical decision-making tool for patients, physicians, hospitals and insurers. For patients, it is a way to gain an additional opinion on a diagnosis, treatment or prognosis from another physician [1]. Physicians seeking another colleague's opinion may refer a patient to another consultant to gain further advice. Many health insurers mandate SO programs to reduce medical costs and eliminate ineffective or sub-optimal treatments [2,3]. Hospitals may also require second reviews as part of routine pathology, radiology reviews or for legal purposes. consultant to consultant referrals. Patients in primary care may also request an opinion from a second specialist when unhappy with the opinion from the first specialist.

The clinical impact of insurer-initiated or hospital-initiated second reviews on diagnosis is well documented [4–8]. The value of SOs in pathology and radiology is also well documented, with improvements in the quality of care and reductions in the rate of diagnostic error firmly established [5–8]. The cost-effectiveness of routine and mandatory SO programs has similarly been extensively studied [2,9]. However, the cost-effectiveness of patient-initiated SOs, and the reasons for initiating SOs, currently remain unclear.

In the context of rising pressure on primary and secondary care services, it is important to set up clear mechanisms for patients seeking second opinions in both public and private systems.

As many patients seek a SO before committing to a treatment plan or a surgery, it is important to understand the advantages vs disadvantages of patient-initiated SOs for themselves, physicians, health services and insurers [10–12]. Seeking a SO may benefit patients medically, provided that the SO is of equal or better quality than the first opinion (FO) [13]. Diagnostic errors, thought to occur in 10% to 15% of cases in general medicine, may be reduced as a result, and better treatment may be recommended [14–16]. SOs may also benefit patients psychologically by enabling them take control of their care and by offering reassurance [17]. However, it is possible that many SOs do not yield medical benefits for patients and may critically delay the treatment [13]. Likewise, SOs may result in disappointment, confusion or increased uncertainty for patients. SOs may increase physician workload and might be perceived as signalling a patient's distrust, harming the doctor-patient relationship [17]. The cost-effectiveness of patient-initiated SOs has also been questioned; SOs may be costly if they involve additional consultations and diagnostic testing, or more expensive treatment recommendations [4,17,18]. In contrast, others have argued that SOs may reduce costs by preventing unnecessary treatment [4], which is a the rationale for insurer-mandated SOs.

A previous systematic review aimed to determine the clinical outcomes of patient-initiated SOs in general medical and surgical care , their satisfaction, characteristics and motivating factors for seeking SO [19]. The review reported that a surprising paucity of studies have examined the impact of patient-

For peer review only

initiated SOs. Patients seeking a SO were mostly women with an average age of 54 years and a diagnosis of breast cancer. Generally, patients were satisfied with SOs, which were more often driven by emotional factors than by concern about their own clinical outcomes. Common motivating factors for seeking a SO were having unresolved symptoms and treatment complications, dissatisfaction with their initial doctor, or seeking additional information. Overall, most patients perceived SOs to be valuable, either because of reassurance or the identification of an alternative [19]. Two other systematic reviews focus on SOs in oncology [13,20].

As new evidence has been accumulated since the last review, conducted in 2013 [19], we carried out an updated review. We aimed to summarise evidence on (1) the characteristics and motivating factors of patients who initiate SOs; (2) the impact of patient-initiated SOs on diagnosis, treatment, prognosis and patient satisfaction; and (3) their cost-effectiveness.

Methods

Eligibility Criteria

A systematic review was performed following the Cochrane Handbook for Systematic Reviews of Interventions approach and using the Preferred Reporting Items for Systematic Reviews and Meta-Analyses (PRISMA) statement to report findings [21,22]. A second medical opinion was defined as a situation in which a patient, after getting a medical opinion from one doctor, obtained another opinion from another doctor regarding their diagnosis, treatment, or prognosis. Eligible studies were published in English-language scientific journals with patient-initiated SOs as the focus, which provided quantitative data on their impact on diagnosis, treatment, prognosis or patient satisfaction, described the characteristics or motivating factors of patients who initiated a SO, or analysed the cost-effectiveness of patient-initiated SOs. Studies that evaluated only physician-initiated referrals, mandatory or routine second reviews, SOs for legal reasons, online or over-the-phone SOs, or SOs in specialised domains such as dentistry and psychiatry, were excluded. Case studies, conference abstracts, comments, editorials, books and review articles were excluded.

Information Sources

A systematic literature search of Embase, Medline, PsycINFO and HMIC databases was performed. Search terms were keywords related to 'SO' and 'patient'. The search strategy was: ((second adj2 opinion*) OR (second adj2 consult*)) AND patient*. The search strategy was developed with a specialist research librarian at Imperial College London and was deliberately designed to achieve high sensitivity. Additional records were identified through hand searching (of reference lists of relevant papers). No date restriction was applied. The searches were conducted in December 2019.

For peer review only

Study Selection

The records identified through database searching and hand searching were first de-duplicated. The titles and abstracts of the remaining records were then independently reviewed by two reviewers (AH and BH) to identify those meeting the inclusion criteria. 10% of the reviewed records were reviewed by another author (GG). Finally, the full text of eligible articles was independently reviewed by two reviewers (AH and BH). Eligibility differences throughout screening were reconciled through discussions.

Data Extraction and Quality Assessment

A data extraction form was developed and used to capture data elements. Study quality was assessed by AH, BH and GG using the National Institutes of Health (NIH) Quality Assessment Tool for Observational Cohort and Cross-Sectional Studies, with 14 questions being answered for each study [23]. The NIH National Heart, lung and blood institute Quality Assessment Tool for Observational Cohort and Cross-sectional studies and Case Control studies is an established and widely used quality assessment tool. It was deemed appropriate because all included studies employed an observational study design, to which this quality assessment tool is applicable. The criteria on the NIH Quality Assessment Tool are designed to help researchers focus on the key concepts for evaluating the internal validity of a study.

Data Synthesis and Analysis

Evidence tables were constructed detailing the characteristics, medical specialties, results and quality of the studies. The outcome measures were then summarised.

Patient and Public Involvement

Patients or the public were not involved in the design, or conduct, or reporting, or dissemination plans of this research.

Results

Database searching identified 4,004 records and hand searching identified one additional record (Figure 1). 1,252 records were excluded during deduplication, resulting in 2,753 unique records. Of these, 2,692 were excluded during title and abstract screening, leaving 61 potentially relevant articles. 28 articles were excluded during a full-text review; 33 articles were included in this review.

For peer review only

Study Characteristics

The 33 included articles described patients with cancer (n=17) and other medical domains (n=16) such as ophthalmology, orthopaedics, neurology and gastroenterology (Appendix 1). Studies were performed in the U.S (n=10), Netherlands (n=7), Israel (n=5), Australia (n=2), Germany (n=3), Japan (n=2), Canada (n=2), Hong Kong and Scotland (both n=1). The 33 studies all used an observational design, either cross-sectional (n=30) or cohort (n=3). The sample size ranged between 36 to 208,366. Studies reported on patient characteristics (n=29), patient motivating factors (n=19), patient satisfaction (n=10) and clinical outcome agreement (n=17). Detailed study findings appear in Appendix 2.

For peer review only

For peer review only

For peer review only - http://bmjopen.bmj.com/site/about/guidelines.xhtml

Risk of bias across studies

All studies used an observational design without control patients. All clearly defined their objective, study population, and exposure and outcome measures, and all consistently implemented across all study participants the inclusion and exclusion criteria for participation in the study, and the exposure and outcome measures. However, only 3 studies measured the exposures of interest prior to the outcomes being measured, and only 11 studies measured key potential confounding variables and adjusted them statistically for their impact on the relationship between exposures and outcomes. No studies blinded outcome assessors to the exposure status of participants. No studies evaluated the possibility of the SO having unintended consequences. The sample size of studies was often small, with 23 studies including fewer than 500 participants and 6 including under 100 (Appendix 3).

Patient Characteristics associated with SO Seeking

More females than males had sought a SO: among patients with general medical concerns 52-61% of patients who sought a SO were female [12,25,26,29,38,39,43,47,55]; Three studies conducted in Netherlands, US, Australia reported that among cancer patients 77-87% of patients who sought a SO were female [33,34,52]. Conversely, two studies conducted in Japan and Germany reported that more male cancer patients sought a SO than female [28,39].

Patients with a higher educational level sought SO more frequently [26,30–32,36,37,39,41–43,47,50].Most patients seeking a SO were middle aged. The mean age of patients who sought a SO ranged from 49 to 59 years [24]. The mean age of patients with general medical concerns ranged from 44to 63 years [25,29,38,43,46,51,55].

Seeking a SO was more common in non-religious patients vs. religious patients having cancer in Israel [36], in patients having cancer who were employed in the US [31,42] and in patients with higher income and socioeconomic status [12,26,36,47,50]. SOs were more common among breast cancer patients who had private insurance [31], and among men with localised prostate cancer with private insurance in the US [42]. Two studies reported on geographic residency, more common for those living in central areas in Israel [12] and for those closer to a SO centre in the Netherlands [53]. Patients seeking a SO with breast cancer were more actively involved in decision-making processes in Germany [30]. Patients seeking a SO from orthopaedics had a poorer relationship with their first doctor in the Netherland [53] and those seeking SO in Japan were more anxious and believed they were in poor health [43]. Seeking a second opinion was negatively related to internal locus of control, perceived health status, and wish to know all details of treatment.[49]

For peer review only - http://bmjopen.bmj.com/site/about/guidelines.xhtml

Patient Motivating Factors

The most common reason reported for seeking a SO was to confirm or refute the suggested diagnosis or treatment or [28,29,33,48,52]; where patients disagreed with their doctor on diagnosis, 44.3% sought a SO [24]. 85% of patients seeking a SO reported on poorly defined complaints by their first physician[38], and 79% for a change in treatment [39]. For example, 59% of patients seeking a SO at a neurological clinic hoped for a different diagnosis or treatment than the first opinion [55]. Among orthopaedic patients, 38-40% questioned the first diagnosis or believed it was incorrect [45,53] and 18% sought reassurance about a recommended surgery [35]. 41% of ophthalmology patients sought a SO because their first doctor indicated that no treatment was possible, or that their prognosis was poor [25]. Patients often sought SOs where they disagreed with their doctor on proposed treatments (29% of drug-related disagreements, and 53% of other treatment disagreements) [24].

Patients often sought a SO to get more information related to diagnosis, treatment options and reassurance [40]. Some were seeking a sub-specialist's opinion [45], with the natural wish 'to be seen by the best doctor' [41]. Dissatisfaction with communication with the first doctor ranged from 19% [45] to 51% [53], where some believed that the first physician did not spent enough time with them [48]. Some patients were encouraged by family members or friends to seek a SO [40], or were recommended a certain doctor by family or friends [53].

Patient Satisfaction

Patients were commonly very satisfied with the SO they received. The SO provided them with reassurance of their treatment or diagnosis, gaining comprehensible information about the treatment [28,40], with a compassionate approach addressing their needs [40] and obtaining answers to their concerns [52]. 84% of SO seekers among the general adult population in Israel were satisfied with the SO and 91% preferred the SO over the FO [45]. 95% of patients enrolled in a national SO program in the US were satisfied with the experience and 87% were more confident in their diagnosis or treatment [35]. In a survey conducted in Japan, most patients who obtained a SO reported they better understood their treatment options (93%), their illness (88%) and the risks of their treatment (82%) [39]. SO consultations in neurology received higher scores than the FO consultations across many aspects of satisfaction: patient involvement in the conversation and in decision-making, information and emotional support given [55]. However, during a 2-year follow-up study, overall satisfaction decreased to the same level as before the SO consultation [54]. 21 out of 37 parents of children with cancer in a paediatric haematology oncology department were satisfied with the second opinion they received [36].

Most patients in all studies were satisfied with their SO consultation. Patients reported feeling more knowledgeable and reassured about their diagnosis and treatment [52], and reported their trust in the

For peer review only

attending physician was strengthened by getting a second opinion [28]. Some patients believed that the second doctor communicated better, answering concerns and providing more information (51%), listening more (39%) and being friendlier (41%) [52].

Clinical agreement between the first and SO

Substantial discrepancies between the first and SOs in diagnosis and suggested treatment were reported across the studies. Diagnosis was confirmed in 50% [39] to 57% [35] of cases, clarified in 17% and changed in 13% [39] to 15% [35]. Among women seeking a SO at a uterine fibroid treatment centre, 13.2% of previous diagnoses of uterine fibroids were unconfirmed by the SO [51]. In people who sought a SO for general medical concerns whilst enrolled in a national SO program, diagnosis was confirmed in 56.8% of cases, clarified in 17% and changed in 14.8% [35]. In patients seeking a SO at an eye hospital, there was 67.9% agreement with surgery recommendations between the FO and SO consultations [25]. Changes in both diagnosis and treatment were experienced by 11% [35] to 56% [55] of patients who sought a SO.

Among lung cancer patients, differences were found between the FO and the SO in 9% of diagnoses (17 patients) and in 13% of cancer stage classification (24 patients) and in 37% of therapeutic advice (70 patients). In total, there were 91 discrepancies between the FO and SO, of which 53 (58%) had a potential major impact on survival, morbidity and quality of life [44].

In surgical oncological cases where the second and first opinions could be directly compared, the advice was identical in 68%, there was a major discrepancy in 16% and a minor discrepancy in another 16% [34].

SO treatment recommended for surgical breast cancer deviated from the FO consultation in 20.3% of 54 cases [27]. 35% of 37 parents of children with haematological cancer were advised to change the treatment advised in the FO [36]. However, 56% of breast cancer patients didn't receive a recommendation for surgery either in their FO or SO consultation [37].

SOs received had a substantial impact on patient decision making. For 42% of cancer patients their SO consultation resulted in a change of treatment.[52] 68% of patients with general medical concerns mentioned they would change or partially change the treatment when the SO and FO differed [39].

Cost-effectiveness

No studies were found to report on the cost-effectiveness of patient-initiated SOs.

For peer review only

Discussion

Summary of findings

Women tended to seek SOs more than men. Most patients seeking a SO were middle aged, with a higher educational level. They tended to be employed, have a higher income and socioeconomic status, and have private medical insurance. Patients seeking a SO sought to gain more information about their condition, gain reassurance about their diagnosis or treatment, were dissatisfied with their previous doctor or were encouraged by family members or friends to seek a SO. Seeking SOs in many cases stemmed from dissatisfaction with the information and the communication with the first doctor, where patients felt they were not given the information or reassurance they sought. Most patients were satisfied with their SO consultation, felt more knowledgeable and reassured about their diagnosis and treatment, and reported having more confidence and trust in their second doctor. Patients believed that their SO doctor communicated better, listened more and was friendlier. A considerable proportion of SO consultations yielded a change in diagnosis or treatment, and these discrepancies had potentially major impact on patient outcomes in up to 58.2% of lung cancer cases. Despite the cost-effectiveness of routine and mandatory SO programs having been extensively studied [56–58], we found no studies reporting on the cost-effectiveness of patient-initiated SOs.

Strengths and Limitations

The review offers a broad overview on the topic of SOs and adds to the previous review in terms of breadth and up-to-dateness [19]. We designed a high-sensitivity search strategy, which did not rely on the "referral and consultation" term used in the previous review. This because a second opinion does not necessarily require a referral, and in many healthcare systems there is no gatekeeping for second opinions and patients can contact a physician privately and independently for a second opinion.

Some limitations should be acknowledged. The main challenge in interpreting these findings is in the cohort of studies from different countries and different healthcare systems, where different insurance models are in place. For example, in some countries and under specific insurance schemes, access to SOs is covered by national and private insurers, whereas in other systems, SOs would be out-of-pocket. Comparison between countries is challenging, as there are substantial differences, not just in the country level, as even in the same country there are different healthcare models and insurance models in each country, not to mention cultural differences in attitudes toward second opinions, which play a significant role. Differences in cultures and attitudes towards parallel consultations with different doctors may also affect the findings presented in studies in this review. Likewise, searching

For peer review only

only for articles in the English-language means that we may have missed eligible articles in other languages.

Comparison with Previous Research

The review offers an updated and broad perspective on patient-initiated SOs. A direct comparison is challenging because we used a different search strategy. This review identified an additional 18 studies, 9 of which were published before the previous review [19]. Three studies [59–61] were included in the previous review [19] but not in this review, because they did not refer to purely patient-initiated SOs [59,61], hence the patient behaviour could not be separated from physician-initiated SOs. Another study referred medical nomadism [60], which is an allied but a different to a seeking second opinion, since it also includes seeking multiple opinions from different experts, not necessarily from the same area of expertise.

Both reviews included only observational studies with an absence of data on control patients. Both reviews found no studies which evaluated the possibility of the SO having unintended consequences. Regarding the characteristics of patients who had sought a SO, the previous review reported only that a large proportion of patients seeking a SO were women with an average age of 54 with a diagnosis of breast cancer. The education level of SO seekers ranged from those with less than a high school education to those with a university degree. This review referred to a broad range of factors pertaining to religious belief; employment, income and insurance; geographic residency; preference for involvement in decision-making; relationship with their first doctor; anxiety and beliefs they were in poor health.

We found similar motivating factors of patients compared to the previous review, with the vast majority of motivating factors for both cancer patients and patients with general medical concerns related to gaining more information about their condition, reassurance about their diagnosis or treatment, or dissatisfaction with their previous doctor. Both reviews found most patients in the studies to be satisfied with their SO consultation, however, a cohort study in this review reported that patient satisfaction dropped in the 2 years following the SO consultation to slightly below the satisfaction with the FO consultation. Both reviews found that SOs most typically confirm the original diagnosis or treatment, but that a considerable proportion of SOs yield a change. We also report that some medical specialties experience significantly more or fewer changes in diagnosis or treatment than average, and that changes in diagnosis and treatment have a more significant impact in cancer patients than in patients with general medical concerns. Two other systematic reviews focus specifically on SO in oncology [13,20]. We did not limit to specific medical specialties and so report evidence on SO in all medical domains.

For peer review only

Implications for practice

While SOs usually confirm the original diagnosis or treatment, a considerable proportion of SO consultations yield a change in treatment. Some medical specialties experienced significantly more changes in diagnosis or treatment, and changes in diagnosis and treatment had a more significant impact in cancer patients than in patients with general medical concerns. In specialities where there are often major discrepancies, there is a case to initiate a SO systematically or at least to make patients aware of the option of seeking a SO. Likewise, in cases where patients delay or avoid making a decision about a treatment course, SOs can help reassure and expedite the treatment. SO may benefit patients emotionally, even if they do not result in medical changes.

The fact that patients seeking a SO tended to be more educated patients, with higher income or socioeconomic status, having private insurance and living in central urban areas, raise concerns about inequalities and access to SOs among deprived groups and those living in rural areas, where access to specialists is limited.

While in many cases the SO confirms the FO, from the patient perspective, a change in their diagnosis or a treatment course may have a crucial impact on their lives, particularly in surgical oncology. From the healthcare system or the insurer perspective, changes in diagnosis or treatment, even if they occur in only a portion of patients, may have substantial impact on patient outcomes, rehabilitation, costs and healthcare staff resources. For example, in the NHS in England, there is a legal requirement that every histopathology assessment should be by two pathologists, which is also built in the health system costing.

SOs stemming from unsatisfactory communication with the first doctor could be potentially avoided by improved doctor-patient communication, offering a detailed explanation and a listening approach. Rather than the SOs being sought confidentially, to not offend the first doctor, doctors should encourage a SO if they sense the patient is in doubt and assist in referring the patient to a suitable consultant and help to come to a mutual decision based on a discussion between the patient and both doctors. By negotiating a treatment that is acceptable to all parties, patients may be spared the confusion associated with discrepant opinions. By preparing patients for the various potential positive and negative outcomes of a SO, doctors can help them make an informed decision about pursuing the SO.

More people taking SO in national healthcare systems will put additional strain on the secondary care, but if unnecessary surgery is cancelled following a SO this will release resources, not to mention a long rehabilitation process which often follow surgery.

For peer review only

Future Research

Although our review suggests that patients generally believe SOs to be valuable, studies infrequently presented follow-up data on patient outcomes. It would also be useful to further explore the extent to which patients are referred back to their initial doctor, and to what extent SOs actually changed the course of treatment (rather than the mere fact that an additional opinion had been obtained). There is a distinct lack of studies on the cost-effectiveness of patient- initiated SOs, despite extensive literature on the cost-effectiveness of routine and mandatory SO programs. Long-term outcomes and potential unintended consequences of SOs must also be examined. Likewise, there is a lack of a uniform definition or objective measures of 'SO', which makes the comparison of findings across studies and health systems challenging. Development of uniform measures will be useful to uniformly compared findings across different countries and healthcare systems. The health systems and related insurance models' aspects, while highly relevant, warrant a broader discussion which was beyond the remit of this review.

Conclusions

We identified demographic characteristics associated with seeking a second opinion, related to age, gender, education, socioeconomic status, place of residence and health condition. Patients seeking a second opinion sought to gain more information or reassurance about their diagnosis or treatment. While many second opinions confirm the original diagnosis or treatment, discrepancies in opinions had a potential major impact on patient outcomes. Research is needed to examine cost-effectiveness of second opinions and to identify patient groups that are likely to benefit from a second opinion. In the context of rising pressure on primary and secondary care services, it is important to set up clear mechanisms for patients seeking second opinions in both public and private systems.

For peer review only

Contributorship statement

GG, LS and BH were involved with conception and design, conducted the data analysis, and drafted the manuscript. AH and HQY were involved in designing and conducting the literature searches, screening, data extraction and synthesis, and revised various versions of the manuscript. ND, JP, SR and AM were involved in conception and design, interpretation of the findings, provided clinical perspectives, and revised various versions of the manuscript.

Data sharing statement

All data are publicly available.

Funding

This report was supported by the National Institute for Health Research Applied Research Collaboration Northwest London, Award Number NIHR200180.

Disclaimer

This article presents independent research commissioned by the National Institute for Health Research (NIHR) under the Applied Health Research (ARC) programme for North West London. The views expressed in this publication are those of the author(s) and not necessarily those of the NHS, the NIHR or the Department of Health.

Ethics

The study did not require an Ethics approval.

Competing Interest

None declared.

For peer review only

Figure legend

Figure 1: PRISMA Flowchart

For peer review only

References

1 Tam KF, Cheng DKL, Ng TY, *et al.* The behaviors of seeking a second opinion from other health-care professionals and the utilization of complementary and alternative medicine in gynecologic cancer patients. *Support Care Cancer Off J Multinatl Assoc Support Care Cancer* 2005;**13**:679–84. doi:10.1007/s00520-005-0841-4

2 Rosenberg SN, Gorman SA, Snitzer S, *et al.* Patients' reactions and physician-patient communication in a mandatory surgical second-opinion program. *Med Care* 1989;**27**:466–77.

3 Odeyemi IA, Nixon J. The role and uptake of private health insurance in different health care systems: are there lessons for developing countries? *Clin Outcomes Res CEOR* 2013;**5**:109–18. doi:10.2147/CEOR.S40386

4 Moumjid N, Gafni A, Bremond A, *et al.* Seeking a second opinion: do patients need a second opinion when practice guidelines exist? *Health Policy Amst Neth* 2007;**80**:43–50. doi:10.1016/j.healthpol.2006.02.009

5 Davidov T, Trooskin SZ, Shanker B-A, *et al.* Routine second-opinion cytopathology review of thyroid fine needle aspiration biopsies reduces diagnostic thyroidectomy. *Surgery* 2010;**148**:1294–301. doi:10.1016/j.surg.2010.09.029

6 Allen TC. Second Opinions: Pathologists' Preventive Medicine. *Arch Pathol Lab Med* 2013;**137**:310–1. doi:10.5858/arpa.2012-0512-ED

7 Raab SS, Grzybicki DM, Janosky JE, *et al.* Clinical impact and frequency of anatomic pathology errors in cancer diagnoses. *Cancer* 2005;**104**:2205–13. doi:10.1002/cncr.21431

8 Tomaszewski JE, Bear HD, Connally JA, *et al.* Consensus Conference on Second Opinions in Diagnostic Anatomic Pathology. *Am J Clin Pathol* 2000;**114**:329–35. doi:10.1093/ajcp/114.3.329

9 Moumjid N, Gafni A, Bremond A, *et al.* Seeking a second opinion: do patients need a second opinion when practice guidelines exist? *Health Policy Amst Neth* 2007;**80**:43–50.

10 Sutherland LR, Verhoef MJ. Why do patients seek a second opinion or alternative medicine? *J Clin Gastroenterol* 1994;**19**:194–7.

11 Wagner T, Wagner L. Who gets second opinions? *Health Aff Proj Hope* 1999;**18**:137–45.

12 Shmueli L, Shmueli E, Pliskin JS, *et al.* Second Medical Opinion: Utilization Rates and Characteristics of Seekers in a General Population. *Med Care* 2016;**54**:921–8. doi:10.1097/MLR.0000000000000567

13 Hillen MA, Medendorp NM, Daams JG, *et al.* Patient-Driven Second Opinions in Oncology: A Systematic Review. *The Oncologist* 2017;**22**:1197–211. doi:10.1634/theoncologist.2016-0429

14 Berner et al. ES. Overconfidence as a Cause of Diagnostic Error in Medicine. *Am J Med* 2008;**121**:S2–23. doi:10.1016/j.amjmed.2008.01.001

15 Graber ML. The incidence of diagnostic error in medicine. *BMJ Qual Saf* 2013;**22**:ii21–7. doi:10.1136/bmjqs-2012-001615

16 Singh H, Giardina TD, Meyer AND, *et al.* Types and Origins of Diagnostic Errors in Primary Care Settings. *JAMA Intern Med* 2013;**173**:418–25. doi:10.1001/jamainternmed.2013.2777

For peer review only

17 Axon A, Hassan M, Niv Y, *et al.* Ethical and legal implications in seeking and providing a second medical opinion. *Dig Dis* 2008;**26**:11–7.

18 Mustafa et al. MK. What is the value of patient-sought second opinions? *Eur J Intern Med* 2002;**13**:445. doi:10.1016/S0953-6205(02)00138-3

19 Payne VL, Singh H, Meyer AND, *et al.* Patient-initiated second opinions: systematic review of characteristics and impact on diagnosis, treatment, and satisfaction. *Mayo Clin Proc* 2014;**89**:687–96. doi:10.1016/j.mayocp.2014.02.015

20 Ruetters D, Keinki C, Schroth S, *et al.* Is there evidence for a better health care for cancer patients after a second opinion? A systematic review. *J Cancer Res Clin Oncol* 2016;**142**:1521–8. doi:10.1007/s00432-015-2099-7

21 Cochrane Handbook for Systematic Reviews of Interventions. 2011.https://handbook-5-1.cochrane.org/ (accessed 25 Sep 2019).

22 Moher D, Liberati A, Tetzlaff J, *et al.* Preferred Reporting Items for Systematic Reviews and Meta-Analyses: The PRISMA Statement. *PLoS Med* 2009;**6**:e1000097. doi:10.1371/journal.pmed.1000097

23 Study Quality Assessment Tools | National Heart, Lung, and Blood Institute (NHLBI). Natl. Inst. Health. https://www.nhlbi.nih.gov/health-topics/study-quality-assessment-tools (accessed 25 Sep 2019).

24 Annandale E, Hunt K. Accounts of disagreements with doctors. *Soc Sci Med* 1998;**46**:119–29. doi:10.1016/S0277-9536(97)00149-4

25 Benson WE, Regillo CD, Vander JF, *et al.* PATIENT–INITIATED SECOND MEDICAL OPINIONS: Their Necessity and Economic Cost. *RETINA* 2001;**21**:633.

26 Cecon N, Hillen MA, Pfaff H, *et al.* Why do newly diagnosed breast cancer patients seek a second opinion? – Second opinion seeking and its association with the physician-patient relationship. *Patient Educ Couns* 2019;**102**:998–1004. doi:10.1016/j.pec.2018.12.017

27 Clauson J, Hsieh YC, Acharya S, *et al.* Results of the Lynn Sage Second-Opinion Program for local therapy in patients with breast carcinoma. Changes in management and determinants of where care is delivered. *Cancer* 2002;**94**:889–94.

28 Fuchs T, Hanaya H, Seilacher E, *et al.* Information Deficits and Second Opinion Seeking – A Survey on Cancer Patients. *Cancer Invest* 2017;**35**:62–9. doi:10.1080/07357907.2016.1242012

29 Gologorsky D, Greenstein S. Retrospective analysis of patients self-referred to comprehensive ophthalmology seeking second opinions. *Clin Ophthalmol* 2013;:1099. doi:10.2147/OPTH.S46448

30 Groß SE, Hillen MA, Pfaff H, *et al.* Second opinion in medical encounters − A study among breast cancer patients. *Patient Educ Couns* 2017;**100**:1990–5. doi:10.1016/j.pec.2017.05.038

31 Katz SJ, Janz NK, Abrahamse P, *et al.* Patient Reactions to Surgeon Recommendations About Contralateral Prophylactic Mastectomy for Treatment of Breast Cancer. *JAMA Surg* 2017;**152**:658. doi:10.1001/jamasurg.2017.0458

For peer review only

32 Kurian AW, Friese CR, Bondarenko I, *et al.* Second Opinions From Medical Oncologists for Early-Stage Breast Cancer: Prevalence, Correlates, and Consequences. *JAMA Oncol* 2017;**3**:391. doi:10.1001/jamaoncol.2016.5652

33 Mellink WA, Dulmen AM, Wiggers T, *et al.* Cancer patients seeking a second surgical opinion: results of a study on motives, needs, and expectations. *J Clin Oncol Off J Am Soc Clin Oncol* 2003;**21**:1492–7.

34 Mellink WAM, Henzen-Logmans SC, Bongaerts AHH, *et al.* Discrepancy between second and first opinion in surgical oncological patients. *Eur J Surg Oncol EJSO* 2006;**32**:108–12. doi:10.1016/j.ejso.2005.08.007

35 Meyer AND, Singh H, Graber ML. Evaluation of Outcomes From a National Patient-initiated Second-opinion Program. *Am J Med* 2015;**128**:1138.e25-1138.e33. doi:10.1016/j.amjmed.2015.04.020

36 Mordechai O, Tamir S, Weyl-Ben-Arush M. Seeking a Second Opinion in Pediatric Oncology. *Pediatr Hematol Oncol* 2015;**32**:284–9. doi:10.3109/08880018.2014.987938

37 Morrow M, Jagsi R, Alderman AK, *et al.* Surgeon recommendations and receipt of mastectomy for treatment of breast cancer. *JAMA J Am Med Assoc* 2009;**302**:1551–6.

38 Mustafa MK, Bijl M, Gans ROB. What is the value of patient-sought second opinions? *Eur J Intern Med* 2002;**13**:445–7. doi:10.1016/S0953-6205(02)00138-3

39 Okamoto S, Kawahara K, Okawa A, *et al.* Values and risks of second opinion in Japan's universal health-care system. *Health Expect* 2015;**18**:826–38. doi:10.1111/hex.12055

40 Philip J, Gold M, Schwarz M, *et al.* Second medical opinions: the views of oncology patients and their physicians. *Support Care Cancer* 2010;**18**:1199–205. doi:10.1007/s00520-009-0742-z

41 Radhakrishnan A, Grande D, Mitra N, *et al.* Second opinions from urologists for prostate cancer: Who gets them, why, and their link to treatment: Second Opinions From Urologists for Prostate Cancer. *Cancer* 2017;**123**:1027–34. doi:10.1002/cncr.30412

42 Ramsey SD, Zeliadt SB, Fedorenko CR, *et al.* Patient preferences and urologist recommendations among local-stage prostate cancer patients who present for initial consultation and second opinions. *World J Urol* 2011;**29**:3–9. doi:10.1007/s00345-010-0602-y

43 Sato T, Takeichi M, Hara T, *et al.* Second opinion behaviour among Japanese primary care patients. *Br J Gen Pract J R Coll Gen Pract* 1999;**49**:546–50.

44 Schook RM, Ter Avest MJ, Van Setten CH, *et al.* Lung Cancer Patients Benefit from Second Opinions by Improvement of Diagnosis and Therapy. *Cancer Clin Oncol* 2014;**3**:p43. doi:10.5539/cco.v3n1p43

45 Shmueli L, Davidovitch N, Pliskin JS, *et al.* Seeking a second medical opinion: composition, reasons and perceived outcomes in Israel. *Isr J Health Policy Res* 2017;**6**:67. doi:10.1186/s13584-017-0191-y

46 Shmueli L, Shmueli E, Pliskin JS, *et al.* Second opinion utilization by healthcare insurance type in a mixed private-public healthcare system: a population-based study. *BMJ Open* 2019;**9**:e025673. doi:10.1136/bmjopen-2018-025673

For peer review only

47 Shmueli L, Davidovitch N, Pliskin JS, *et al.* Reasons, perceived outcomes and characteristics of second-opinion seekers: are there differences in private vs. public settings? *BMC Health Serv Res* 2019;**19**:238. doi:10.1186/s12913-019-4067-4

48 Sutherland LR, Verhoef MJ. Patients who seek a second opinion: are they different from the typical referral? *J Clin Gastroenterol* 1989;**11**:308–13.

49 Sutherland LR, Verhoef MJ. Why do patients seek a second opinion or alternative medicine? *J Clin Gastroenterol* 1994;**19**:194–7.

50 Tam KF, Cheng DK, Ng TY, *et al.* The behaviors of seeking a second opinion from other health-care professionals and the utilization of complementary and alternative medicine in gynecologic cancer patients. *Support Care Cancer Off J Multinatl Assoc Support Care Cancer* 2005;**13**:679–84.

51 Tan N, McClure TD, Tarnay C, *et al.* Women seeking second opinion for symptomatic uterine leiomyoma: role of comprehensive fibroid center. *J Ther Ultrasound* 2014;**2**:3. doi:10.1186/2050-5736-2-3

52 Tattersall MHN, Dear RF, Jansen J, *et al.* Second opinions in oncology: the experiences of patients attending the Sydney Cancer Centre. *Med J Aust* 2009;**191**:209–12.

53 van Dalen I, Groothoff J, Stewart R, *et al.* Motives for seeking a second opinion in orthopaedic surgery. *J Health Serv Res Policy* 2001;**6**:195–201.

54 Wieske L, Richard E, Wijers D, *et al.* Long-term satisfaction after neurological second opinions and tertiary referrals. *Eur J Neurol* 2011;**18**:1310–6. doi:10.1111/j.1468-1331.2011.03394.x

55 Wijers D, Wieske L, Vergouwen MDI, *et al.* Patient satisfaction in neurological second opinions and tertiary referrals. *J Neurol* 2010;**257**:1869–74. doi:10.1007/s00415-010-5625-1

56 I. Epstein et al. J, Fred. Clinical and Cost Impact of Second-opinion Pathology. *Am J Surg Pathol* 1996;**20**:851–7. doi:10.1097/00000478-199607000-00008

57 Lindsey PA, Newhouse JP. The Cost and Value of Second Surgical Opinion Programs: A Critical Review of the Literature. *J Health Polit Policy Law* 1990;**15**:543–70. doi:10.1215/03616878-15-3-543

58 Torok CM, Lee C, Nagy P, *et al.* Neuroradiology Second Opinion Consultation Service: Assessment of Duplicative Imaging. *Am J Roentgenol* 2013;**201**:1096–100. doi:10.2214/AJR.12.9429

59 Grafe WR, McSherry CK, Finkel ML, *et al.* The Elective Surgery Second Opinion Program. *Ann Surg* 1978;**188**:323–30.

60 Boudali A, Bahiri R, Hmamouchi I, *et al.* The prevalence of medical nomadism of the followed patients in rheumatology. *Rheumatol Int* 2012;**32**:1639–43. doi:10.1007/s00296-011-1823-0

61 Bekkelund SI, Salvesen R, North Norway Headache Study (NNHS). Are headache patients who initiate their referral to a neurologist satisfied with the consultation? A population study of 927 patients--the North Norway Headache Study (NNHS). *Fam Pract* 2001;**18**:524–7. doi:10.1093/fampra/18.5.524

For peer review only

For peer review only

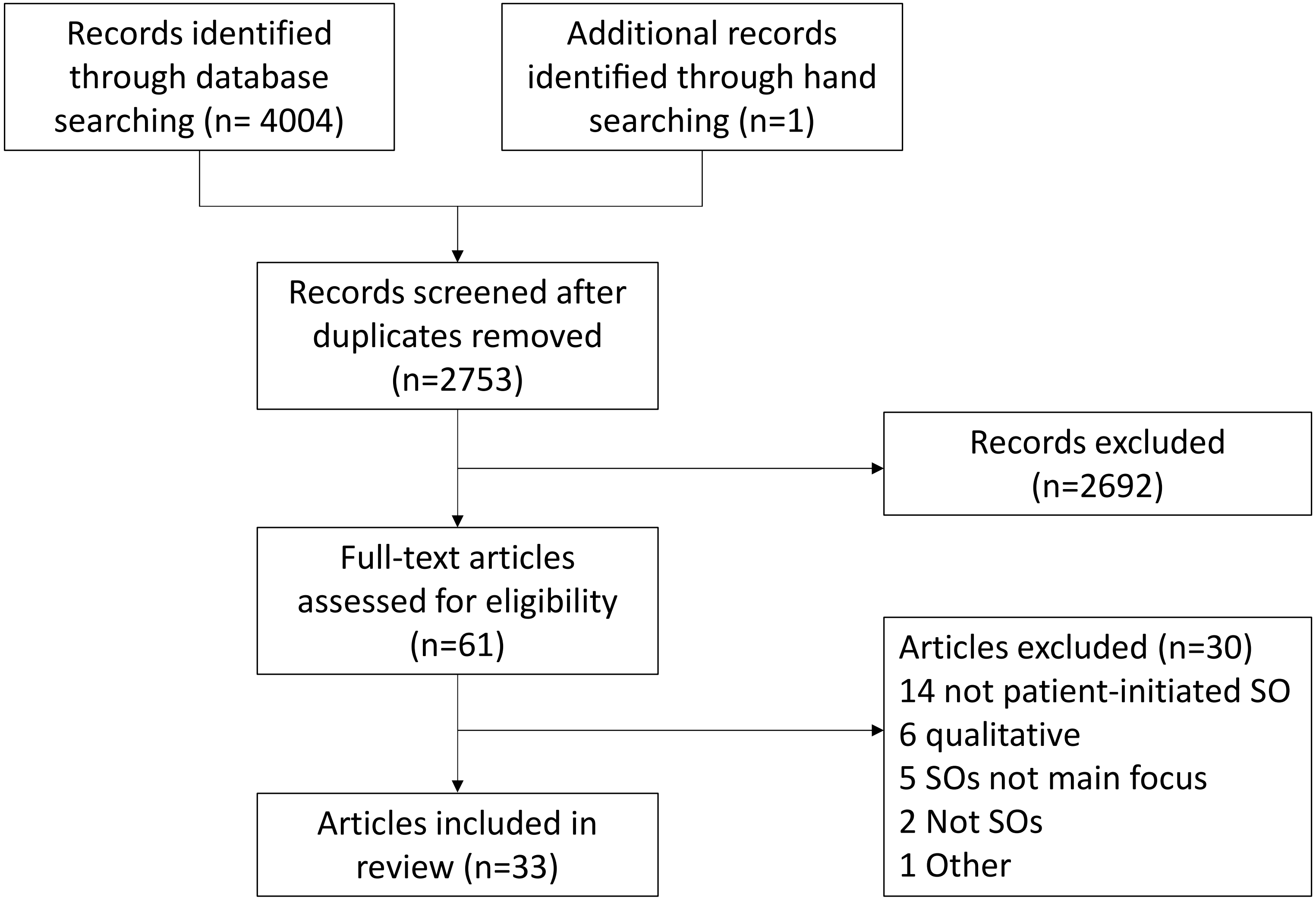
Records identified through database searching (n= 4004)
Additional records identified through hand searching (n=1)
Records screened after duplicates removed (n=2753)
Records excluded (n=2692)
Full-text articles assessed for eligibility (n=61)
Articles excluded (n=30)
14 not patient-initiated SO
6 qualitative
5 SOs not main focus
2 Not SOs
1 Other
Articles included in review (n=33)

Appendix 1: Study characteristics

Study	Medical specialty	Location	Study design	No. of participants	Characteristics	Motivation	Satisfaction	Diagnosis	Treatment	Prognosis
Annandale, 1998[24]	Gynaecology, respiratory, cardiovascular, other	Scotland	Cross-sectional	307		X				
Benson, 2001[25]	Ophthalmology	United States	Cross-sectional	100	X	X			X	
Cecon. 2019[26]	Breast cancer	Germany	Cross-sectional	419	X	X		X	X	
Clauson, 2002[27]	Breast cancer	United States	Cross-sectional	231	X				X	
Fuchs, 2017 [28]	Cancer	Germany	Cross-sectional	36	X	X	X		X	
Gologorsky, 2013[29]	Ophthalmology	United States	Cross-sectional	174	X	X				

For peer review only

Study	Medical specialty	Location	Study design	No. of participants	Characteristics	Motivation	Satisfaction	Diagnosis	Treatment	Prognosis
Groß, 2017[30]	Breast cancer	Germany	Cross-sectional	2846	X	X				
Katz, 2017[31]	Breast cancer	United States	Cross-sectional	304	X					
Kurian, 2017[32]	Breast cancer	United States	Cross-sectional	168	X					
Mellink, 2003[33]	Cancer	Netherlands	Cross-sectional	212	X	X				
Mellink, 2006[34]	Cancer	Netherlands	Cohort	403	X			X	X	X
Meyer, 2015[35]	Orthopaedics, oncology, haematology, other	United States	Cross-sectional	6791		X	X	X	X	
Mordechai, 2015[36]	Haematological cancer	Israel	Cross-sectional	37	X		X		X	

For peer review only

Study	Medical specialty	Location	Study design	No. of participants	Characteristics	Motivation	Satisfaction	Diagnosis	Treatment	Prognosis
Morrow, 2009[37]	Breast cancer	United States	Cross-sectional	378	X				X	
Mustafa, 2002[38]	Fatigue, abdominal pain, chest pain, other	Netherlands	Cross-sectional	201	X	X		X	X	
Okamoto, 2013[39]	Cancer, neurology, orthopaedics, other	Japan	Cross-sectional	149	X	X	X	X	X	
Philip, 2010[40]	Cancer	Australia	Cross-sectional	17/65*	X	X	X			
Radhakrishnan, 2017[41]	Prostate cancer	United States	Cross-sectional	950	X	X				
Ramsey, 2011[42]	Prostate cancer	United States	Cohort	143/25*	X				X	
Sato, 1999[43]	Obstetrics, gynaecology,	Japan	Cross-sectional	420	X					

Study	Medical specialty	Location	Study design	No. of participants	Characteristics	Motivation	Satisfaction	Diagnosis	Treatment	Prognosis
	gastroenterology, other									
Schook, 2014[44]	Lung cancer	Netherlands	Cross-sectional	184	X			X	X	
Shmueli, 2016[12]	Orthopaedics, ophthalmology, dermatology, other	Israel	Cross-sectional	208,366	X					
Shmueli, 2017[45]	Orthopaedics, ophthalmology, dermatology, other	Israel	Cross-sectional	344		X	X	X	X	
Shmueli. 2019 [46]	Orthopaedics, ophthalmology,	Israel	Cross-sectional	143,371	X					

For peer review only

Study	Medical specialty	Location	Study design	No. of participants	Characteristics	Motivation	Satisfaction	Diagnosis	Treatment	Prognosis
	dermatology, other									
Shmueli. 2019a[47]	Orthopaedics, ophthalmology, dermatology, other	Israel	Cross-sectional	339	X	X	X	X	X	
Sutherland. 1989 [48]	Gastroenterology	Canada	Cross-sectional	246	X	X				
Sutherland. 1994 [49]	Gastroenterology	Canada	Cross-sectional	341	X					
Tam, 2005[50]	Gynaecologic cancer	Hong Kong	Cross-sectional	80	X	X				
Tan, 2014[51]	Gynaecology	United States	Cross-sectional	205	X			X	X	

For peer review only

Study	Medical specialty	Location	Study design	No. of participants	Characteristics	Motivation	Satisfaction	Diagnosis	Treatment	Prognosis
Tattersall, 2009[52]	Cancer	Australia	Cross-sectional	77	X	X	X		X	
Van Dalen, 2001[53]	Orthopaedics	Netherlands	Cross-sectional	401-411/349*	X	X				
Wieske, 2011[54]	Neurology	Netherlands	Cohort	76			X			
Wijers, 2010 [55]	Neurology	Netherlands	Cross-sectional	183	X	X	X	X	X	

* indicates first doctors of patients who also participated in the study

For peer review only

Appendix 2: Detailed Study findings

Study	Participants	Study aim	Characteristics	Diagnosis/Treatment/Prognosis	Motivation/Satisfaction
Annandale, 1998[24]	Community sample of individuals	To explore doctor-patient disagreements, disagreement actions, and the relationship between them			**Motivation:** SO sought in 52.3% of diagnosis-related disagreements, 28.6% of prescribed drug-related disagreements, 53.3% of other treatment-related disagreements, 34.5% of disagreements where the patient felt the health problem had not been taken seriously, 33.3% of disagreements centred on the doctors' interactional style and 45.5% of other disagreements
Benson, 2001[25]	Patients seeking an SO at an eye hospital	To assess the value of patient initiated SOs for patients and third-party payers	56% female, mean age 63 years, median age 66 years, 39% college-level education or higher, 39% employed, mean travel distance 42.5 miles, median travel distance 20 miles, 87% thought their insurer would pay	**Treatment:** 67.9% agreement with surgery recommendations, 41.7% agreement with laser treatment recommendations, 81.8% agreement with vitrectomy recommendations, and 100% agreement with scleral buckling procedures, cataract surgery and extruding scleral buckle removal.	**Motivation:** 41% sought an SO primarily because their first physician indicated that no treatment was possible or that even with treatment, the prognosis was poor. 20% wanted a better explanation of their problems, 9% specifically wanted a specialist from the hospital, 7% wanted an SO before surgery, 6% were not making progress

For peer review only - http://bmjopen.bmj.com/site/about/guidelines.xhtml

			for the SO	Major disagreement in 8.3% of cases for which no surgery had been recommended	with their current treatment, 6% were encouraged by a friend or family, 4% did not like their first physician, 3% wanted a specialist, 2% were encouraged by a family physician, and 2% believed that they were being pushed into treatment
Cecon. 2019[26]	Newly diagnosed breast cancer patients from 86 hospitals in Germany completed a postoperative mail survey	To examine breast cancer patients' reasons to seek a second opinion (SO) and the underlying variables. To find out more about the outcome of the SO, the perceived helpfulness and the effect on the physician-patient relationship.			Reasons to seek an SO were mostly unrelated to the physician-patient relationship. Reasons related to the physician-patient-relationship were associated with a lower education level.<br>A different treatment plan recommendation (25%) reportedly affected the patients' relationship with their primary physician.
Clauson, 2002[27]	Breast cancer patients (stage I, II or intraductal carcinoma) seeking a	To determine how often a SO on the local therapy of breast carcinoma changed patient	Mean age 51.4 years, 89% Caucasian, 70% more than a high school education, 80%	**Treatment:** The SO differed from the first opinion (FO) in 20.3% of cases	

For peer review only

	second surgical opinion at a breast centre	management, and to identify factors predictive of remaining at the SO site for therapy	employed outside the home		
Fuchs, 2017 [28]	Cancer patients who participated in a series of lectures held by a regional cancer society on complementary and alternative medicine (CAM)	To explore cancer patients' motivation for seeking an SO	Males sought SOs more than females (79% males vs 53% females). Patients who reported low understanding of information sought an SO more often	**Treatment:** 66.7% of patients remained the same	**Motivation:** 80.6% wanted to check the correctness of treatment. 48.6% wanted to gain a better understanding of their diagnosis, with a positive correlation between this desire and experiencing a higher gain of information after an SO, and with this desire playing a stronger role in the decision to seek an SO in males than females<br>**Satisfaction:** 56.3% stated their trust in the attending physician was strengthened by getting an SO, with those patients feeling a high degree of satisfaction with the information about their planned treatment and the effects of the prescribed pharmaceuticals. 78.7% felt assured afterwards, with those patients feeling significantly less burdened by the disease

For peer review only

Gologorsky, 2013[29]	Patients that self-referred to an ophthalmology clinic seeking an SO	To determine the reasons that patients self-refer to an ophthalmology clinic seeking an SO	57.5% female, mean age 60.9 years		**Motivation:** 59.8% requested confirmation of diagnosis or more information, with 54% coming from outside ophthalmologists and 5.7% from outside optometrists. 40.2% had suffered a previous adverse experience with an outside medical provider, with 25.9% perceived treatment failure or complications, 6.9% poor provider communication skills, 4.6% distrust of provider and 2.9% poor bedside manner
Groß, 2017[30]	Newly diagnosed breast cancer patients with at least one postoperative histological finding of breast cancer, who underwent surgery in a breast cancer centre hospital	To examine the association between whether physicians discuss the possibility of seeking an SO with patients and the patients' decision to seek an SO, as well as the impact of seeking such an opinion on patients' trust in physicians	Patients informed about the possibility of requesting an SO, patients more actively involved in the decision-making process and patients with a school-leaving certificate were more likely to seek an SO. The better the information provided by doctors as reported by		**Motivation:** Patients requesting an SO were more likely to not trust their physician. Patients aged between 18 and 66 years had less trust in their doctor than patients older than 75 years. The better the information provided by the doctor and the more patients were involved in the decision-making process, the higher the likelihood of patients indicating they had a trusting doctor-patient relationship

For peer review only

			patients, the lower the likelihood of seeking an SO		
Katz, 2017[31]	Breast cancer patients (stage I, II or intraductal carcinoma) who had received surgery and had considered contralateral prophylactic mastectomy (CPM) with their first surgeon	To examine the association between patient report of first surgeon recommendation against CPM and the extent of discussion about it with 3 outcomes: patient satisfaction with surgery decisions, receipt of second opinion, and receipt of surgery by a second surgeon	SOs more common among patients who were younger, more educated, did not have Medicare health insurance and who worked for pay. Women who received a recommendation against CPM were not more likely to seek an SO (17.1% among patients with recommendation against CPM vs 15% among others)		
Kurian, 2017[32]	Breast cancer patients (stage I, II or intraductal	To investigate the patterns and correlates of SO use, and their impact	Receiving a SO was significantly associated with a college education		

For peer review only

	carcinoma) who had received surgery	on chemotherapy decisions and communication with oncologists	vs less education, a higher preference for making one's own treatment decisions vs a lower preference, and frequent use of internet-based support vs no use		
Mellink, 2003[33]	Cancer patients seeking an SO at a surgical oncology outpatient clinic	To explore the sociodemographic and clinical characteristics of cancer patients seeking an SO consultation, and to analyse their SO-related motives, needs and expectations	81.6% female, mean age 53 years, 50.5% less than a high school education	The mean score on information need was 3.4 about the disease, 3.7 about the treatment and 3.5 about the prognosis and expected course. Hope for a difference between the first and second opinion was expressed by 68% of the patients, whereas 22% hoped for identical advice	**Motivation:** With a range from 1 (not at all) to 4 (a lot), the mean score on internal motivation (associated with the need for reassurance and more certainty) was 3.66. The mean score on external motivation (related to negative experiences or unfulfilled needs) was 2.48. Externally motivated patients more often hoped for different advice. Patients with non-metastatic disease, a high level of anxiety disposition and preference for an active role in decision-making were relatively more often externally motivated.

For peer review only

Mellink, 2006[34]	Cancer patients seeking an SO at a surgical oncology outpatient clinic	To prospectively describe in a population of oncological SO patients the outcome of routine revisions of histopathological and radiological material, the frequency and extent of discrepancy between the second and first opinion, and the location of further treatment or follow-up	87.3% female, mean age 52 years	**Diagnosis/Treatment/Prognosis:** Major difference in diagnosis, treatment or prognosis was identified in 16.4% of patients, minor difference in 15.5% and no difference in 68.1%. Pathology review resulted in a difference which affected prognosis or therapy in 3.4% of cases and a difference not affecting prognosis or therapy in 2.8%. Radiology review resulted in a difference affecting prognosis or therapy in 1.6% of cases and a difference not affecting prognosis or therapy in 2.8%	
Meyer, 2015[35]	Patients who sought an SO whilst enrolled in a national SO program allowing employee-beneficiaries to request free SOs	To examine the outcomes of SOs provided by a national patient-initiated SO program		**Diagnosis:** 56.8% cases confirmed, 17% clarified, and 14.8% changed. Anaesthesiology, gastroenterology, neurology, and rheumatology resulted in significantly more changes than average. Cardiovascular disease,	**Motivation:** 41.3% needed help choosing treatment options, 22.5% had symptoms that were not improving, 18% were questioning whether to proceed with recommended surgery, 6.3% sought a diagnosis, 6% did not understand their diagnosis, and 6% were sceptical of their

For peer review only

	from expert specialists			medical oncology and haematology, surgical oncology, and urology resulted in significantly fewer. Minor clinical impact in 46.3% of cases, moderate impact in 18.2% of cases, and major impact in 2.7% of cases. Critical care/pulmonary medicine, gastroenterology, infectious diseases, neurology, and obstetrics and gynaecology resulted in significantly more estimates of moderate/major clinical impact than average. General surgery, ophthalmology, and radiation oncology resulted in significantly fewer<br>**Treatment:** 26.4% cases confirmed, 26.9% clarified, and 37.4% changed. Allergy and immunology, anaesthesiology, gastroenterology, neurological surgery, obstetrics and	physician<br>**Satisfaction:** 94.7% were satisfied with the SO experience, 89.6% had their questions answered and 87.3% were more confident in their diagnosis or treatment choice afterwards

For peer review only

				gynaecology, otolaryngology, physical medicine and rehabilitation, and rheumatology resulted in significantly more changes than average. General surgery, medical oncology and haematology, surgical oncology, and urology resulted in significantly fewer. Minor clinical impact in 50.1% of cases, moderate impact in 26.5% of cases, and major impact in 4.2% of cases. Colon and rectal surgery, medical oncology and haematology, obstetrics and gynaecology, and thoracic surgery resulted in significantly more estimates of moderate/major impact than average. Cardiovascular disease, general surgery, internal medicine, neurology, ophthalmology, and physical medicine and	

For peer review only

				rehabilitation resulted in significantly fewer **Diagnosis/Treatment:** 10.6% of cases had changes in both diagnosis and treatment	
Mordechai, 2015[36]	Parents of children with cancer recently treated in a paediatric haematology oncology department	To investigate the epidemiology and motivations of the families who sought an SO	More common for those with a higher socioeconomic status, those with a higher number of educational years and those more non-religious	**Treatment:** 35.1% were advised to change their therapy	**Satisfaction:** 56.7% were satisfied with the second opinion, 29.7% found it was not effective and 24.3% found it unsettling
Morrow, 2009[37]	Breast cancer patients (stage I, II or intraductal carcinoma)	To evaluate the association of patient-reported initial recommendations by surgeons and those given if an SO was sought with receipt of initial mastectomy, and to assess the use of	**Characteristics:** More common for woman with a higher educational level and those initially advised to undergo mastectomy	**Treatment:** 12.1% received a discordant opinion from a second surgeon. 20.2% of patients who received an initial mastectomy recommendation received an SO for BCS. 11.9% of patients who received an initial BCS recommendation received an SO for mastectomy. 56.5% of patients	

For peer review only

		mastectomy after attempted breast-conserving surgery (BCS)		who did not receive a first surgeon's recommendation similarly did not receive one from a second surgeon	
Mustafa, 2002 [38]	Patients with chronic unresolved symptoms or treatment issues seeking an SO in a general medicine outpatient clinic	To explore the nature and possible benefits of patient-generated SOs in general internal medicine	58.2% female, mean age 46 years	**Diagnosis:** When an SO was sought for diagnostic reasons, a definite diagnosis was established in only 10% of cases<br>**Treatment:** When an SO was sought for therapeutic advice, a useful new treatment plan was obtained in 71% of cases	**Motivation:** 84.6% had poorly defined complaints that could not be satisfactorily explained or diagnosed by their original physician, and 15.4% sought management advice (3% sought better control of their blood pressure and 1.5% sought better control of their diabetes)
Okamoto, 2013[39]	Patients in the SO clinic (group A) and general patient waiting area (group B) of a university hospital	To investigate the characteristics and motivation of patients who seek SOs in Japan's universal healthcare system, and to explore how these SOs affect understanding and management	51.7% female, 56.2% 40-64 years and 70.5% no medical provider in the family. 54.1% had a 4-year college education or higher. Those who finished graduate school were 9.5 times, and those who completed 4-year college were 2.1 times more likely to	**Diagnosis/Treatment:** 8.8% of SOs were the same, 41.5% were almost the same, 27.2% were partially different, and 12.9% were different<br>**Treatment:** 17.7% of patients would not ask to change their treatment plan as a result of the SO, 10.2% would be unlikely to ask, 23.1% would ask to partially change, and 22.4% would ask to	**Motivation (group A):** 100% believed an SO would be sought for better understanding, 97% believed for decision-making, 77.6% believed for changing ongoing treatment, and 50.7% believed for changing doctor<br>**Motivation (group B):** 92.6% believed an SO would be sought for better understanding, 95.1% believed for decision-making, 84.1% believed for changing ongoing treatment, and 67.9%

			obtain an SO than those with a high school education or lower	change**.** When the SO was the same or almost the same as the FO, 25.7% would seek a change or partial change. When the SO and FO differed, 67.8% would seek a change or partial change	believed for changing doctor<br>**Satisfaction (group A):** 92.5% better understood treatment options, 87.9% better understood their illness and plan, 87.7% better understood that their treatment was specifically designed for their health condition, 81.8% better understood the risks of their treatment, and 81.5% better understood uncertainty in medicine<br>**Satisfaction (group B):** 81.5% better understood treatment options, 77.8% better understood the risks of their treatment, 73.2% better understood their illness and plan, 66.7% better understood that their treatment was specifically designed for their health condition, and 61.3% better understood uncertainty in medicine
Philip, 2010[40]	Advanced cancer patients attending specialist clinics in a	To explore the views on SOs held by advanced cancer patients and their	**According to group B:** 84% characterised SO patients (SOPs) as		**Motivation (group A):** 26.8% of reasons given related to concerns around communication, 32.1% related to the

For peer review only

	quaternary hospital (group A) and their FO medical oncologists (group B)	medical oncologists, including motivation, satisfaction and the impact they may have on the doctor-patient relationship	having greater information needs, 58% believed they had greater psychosocial needs, and 77% believed they took more physician time and energy than the overall patient population		extreme and desperate nature of the situation, 12.5% related to the need for reassurance, 12.5% related to concerns with care, and 8.9% related to SOs being prompted by other parties including family, friends or as result of information in the media<br>**Motivation (according to group B):** 75% of reasons suggested related to a need for additional information, 70% related to family or friends urging an SO, 70% related to a need for reassurance regarding diagnosis and treatment course, 60% related to a need for communication in a different form, 60% related to a need for information in a different form, 53% related to a need to leave 'no stone unturned', 51% related to a need for different style or personality in the doctor, 48% related to the patient requesting more treatment, 37% related to reassurance offered by the public status of the doctor or their

For peer review only

					institution, and 14% related to an SO being akin to getting quotes<br>**Satisfaction (group A):** 94.1% found the SO helpful, with 44.2% of responses relating satisfaction to the communication or manner of the second doctor and 38.4% relating satisfaction to reassurance<br>**Satisfaction (according to group B):** 91% considered reassurance to be important to patient satisfaction, 83% suggested different or more comprehensive information, 83% suggested an approach to care which was more commensurate with the patient's needs, 69% suggested the skills of the doctor, 57% suggested the different organisation of time within the consultation, and 51% suggested the status of the doctor to be important
Radhakrishnan, 2017[41]	Newly diagnosed local-stage prostate	To assess the frequency of and reasons for SOs for local-stage prostate	Younger men and men with college-level education or higher were		**Motivation:** 50.8% wanted more information about their cancer (younger men and men with a college-level

For peer review only

	cancer patients	cancer and the characteristics of the patients who seek them, and to assess whether SOs are associated with treatment choice and perceived quality of prostate cancer care	more likely to obtain an SO		education or higher more likely to), 46.3% wanted to be seen by the best doctor (younger men more likely to), 31% were encouraged by a family member or friend to obtain an SO, 25% wanted to find out about treatment not offered by their first doctor, and 15.5% were dissatisfied with their initial urologist (patients aged 75 years or older least likely to)
Ramsey, 2011[42]	Newly diagnosed local-stage prostate cancer patients and their urologists at academic urology clinics	To compare patient preferences and urologist recommendations for treatment among local-stage prostate cancer patients presenting for initial management consultations versus SOs	Men seeking SOs were significantly younger, more educated, more likely to have private insurance and more likely to be employed. 53.8% had low-risk disease and 23.1% listed two or more non-cancer comorbidities at diagnosis	**Treatment:** Prostatectomy was the dominant treatment recommended at SO visits, with less than 20% of urologists reporting recommending other options. During initial consultations, other treatments were more likely to be recommended in addition to prostatectomy. SO consultations associated with a fewer number of treatment recommendations (0.52 fewer)	

For peer review only

Sato, 1999[43]	Primary care patients in the general medicine clinic of a university hospital	To describe the sociodemographic characteristics of SO patients and to determine the factors related to this behaviour	60.5% female, mean age 45.2 years, 62.6% married, 88.3% employed. SOPs had a significantly higher educational level than doctor-shopping patients (DSPs), with 78.8% having more than nine years of education. General Health Questionnaire (GHQ) scores of SOPs were significantly higher than those of first opinion patients (FOPs), suggesting that SOPs are more anxious. Compared with FOPs, SOPs were more likely to have a chronic illness and to believe they were in poor health, also taking		

For peer review only

			more advice from anybody. DSPs were more likely than SOPs to believe they were in poor health, to mistrust their diagnosis and treatment, and to have high expectations for the hospital. FOPs had a significantly higher frequency of diagnosis for endocrinological and metabolic disorders than did SOPs. SOPs had a significantly higher frequency of diagnosis for obstetric and gynaecological disorders than did DSPs. DSPs had the most frequent diagnosis of psychiatric illnesses compared with FOPs and SOPs		

For peer review only

Schook, 2014[44]	Lung cancer patients referred to a specialist pulmonary oncology outpatient clinic	To collect data from the initial evaluation of patients referred to the clinic for an SO and compare these with the data of the SO conducted to identify discrepancies in diagnosis, stage and therapeutic advice	57.4% male, mean age 59 years	**Diagnosis/Treatment:** There were discrepancies for 9% of diagnosis, 12.8% of stage, and 37.2% of therapeutic advice. 58.2% of patients with discrepancies had a potential major impact on patient outcomes, 21.9% had a potential minor impact and 19.8% had a potential identical impact	
Shmueli, 2016[12]	Active members of the largest regional health fund who visited at least one specialist within a 20-month period (group A) plus a representative random sample of the general adult population (group B)	To estimate how many people seek SOs and to determine the characteristics of SO seekers	**Group A:** More women than men, native-born and established immigrants than recent immigrants, older people than younger people, people in high and middle socio-economic levels than low income level, people living in central areas and intermediate localities than people living in		

For peer review only

			peripheral areas, and people with chronic conditions than people with no chronic conditions<br>**Group B:** More women than men, native-born and established immigrants than recent immigrants, and people living in central areas and intermediate localities than people living in peripheral areas. Those who perceived their health as good or very good sought fewer SOs than those who perceived their health as not so good		

Shmueli, 2017[45]	Representative random sample of the general adult population	To characterise SO seekers, their reasons for seeking an SO and choosing a specific physician, and their perceived outcomes following the SO		**Diagnosis/Treatment:** 56.1% mentioned there was a difference in diagnosis or treatment between the FO and SO	**Motivation:** 38.1% stated they wanted to verify their diagnosis with another doctor or they had doubts about the treatment recommended, 19.4% were seeking a sub-specialist's opinion, 19.2% were dissatisfied with communication with their first doctor or felt they didn't receive enough information, and 15.4% believed previous treatments were ineffective<br>**Satisfaction:** 84.3% were satisfied with the SO, 91% preferred the SO over the FO and 76.5% experienced health improvement after the SO
Shmueli. 2019 [46]	Patients aged 21 years and above who visited at least one specialist over an 18 months period, either in the secondary care or privately via the	To evaluate the utilization (overall and by specialty) and the characteristics of second-opinion seekers by insurance type (either health fund or	second-opinion seekers via the health fund tended to be females, of age 40–59 years and with chronic conditions. In contrast, second-opinion seekers via the supplementary insurance tended to be native-born		

For peer review only

	supplementary insurance	supplementary insurance) in a mixed private-public	and established immigrants, in a high socioeconomic level and living in central areas.		
Shmueli. 2019a[47]	Representative random sample of the general adult population	To evaluate (1) the characteristics of people seeking SOs in the private system vs. the public system in Israel; (2) the reasons for seeking private SOs; and (3) the perceived outcomes of SOs given in a private system vs. a public system	Low self-reported income group, immigrants (immigrated to Israel after 1989) and religious people tended to seek SOs from the public system more than others.		The main reason for seeking a SO from a private physician rather than from the public system was an assumption that private physicians are more professional (n = 58, 45.7% of 127). The other reasons were prior acquaintance with the physician or a word-of-mouth about the specific private physician (n = 21, 16.5%), waiting time at the health fund (n = 18, 14.2%), that private physicians have better attitudes (n = 13, 10.2%) or other reasons such as flexible hours, restrictions of the public health fund, etc.
Sutherland. 1989 [48]	Patients referred for the first time to a university-based gastrointestinal unit.	To investigate how many of the patients, referred for the first time, were seeking a second opinion-	Patients who sought a second opinion were more apt to have symptoms for >2 years,		Main reasons for seeking a SO These patients either (a) believed that the original gastroenterologist had not spent

		-i.e., a second consultation within 2 years of seeing a gastroenterologist.	perceive their health to be fair to poor, fewer than two have seen general practitioners in the past year, have spent >6 days in hospital in the last year.		enough time with them or (b) wanted a confirmation of the original diagnosis.
Sutherland. 1994 [49]	Patients attending a university-based gastroenterology clinic were asked to complete a self-administered questionnaire.	To determine psychosocial determinants of two measures of health care use: seeking a second opinion and alternative medicine use, and to assess whether changes in these two measures of health care use had taken place during the past 4 to 5 years.	Seeking a second opinion was negatively related to internal locus of control, perceived health status, and demanding to know all details of treatment. Eight percent (28) of all patients saw an alternative practitioner for the same problem for which they saw the gastroenterologist compared to 9% 4 years ago.		

For peer review only

Tam, 2005[50]	Gynaecologic cancer patients attending a gynaecologic cancer clinic of a tertiary referral (TR) centre	To determine the prevalence and predictors for seeking an SO and the utilisation of CAM among gynaecologic cancer patients, and the relationship between the two behaviours	Mean age 48.7 years. More likely to seek an SO if late-stage disease, previous treatment with radiotherapy, tertiary education or income >US$30,000. 71.3% had used CAM, being 2.47 times more likely than non-SO seekers		**Motivation:** 45.7% of patients had complications or side effects arising from the standard cancer treatment, 37% just wanted to see more doctors, and 17.4% wanted some advice to maintain a better 'well-being'. Patients who gave a positive answer to 'I am doubtful to what my doctors have done on me' or 'I would receive better care if I see more doctors' were more likely to seek an SO
Tan, 2014[51]	Women seeking an SO for management of symptomatic uterine fibroids at a multidisciplinary uterine fibroid treatment centre in a tertiary care facility	To describe the early experience of a comprehensive uterine fibroid treatment centre and report results in women seeking an SO for management of symptomatic uterine fibroids	Mean age 43.8 years, 79% had not had prior therapy	**Diagnosis:** Nearly all had received a diagnosis of uterine fibroids from outside clinics but only 86.8% were found to have them<br>**Treatment:** Most had been offered hysterectomy from outside facilities. Medical therapy or no further treatment was recommended for 22% of	

For peer review only

				patients. 77.9% underwent procedures and 7.3% underwent hysterectomy. 53.2% elected to transfer their care to the treatment centre	
Tattersall, 2009[52]	Cancer patients seeking an SO in a medical oncology clinic	To investigate the frequency, goals and outcomes of SO consultations in a medical oncology clinic	**Characteristics:** 76.6% female, median age 55 years, 68.8% only English spoken at home, 85.7% married, 89.6% no medical or allied health training, 33.8% had a university degree, 68.8% had started treatment recommended by their first oncologist more and 0% believed less, 35.3% believed SO doctor seemed more knowledgeable and 2% believed less, 51% believed SO doctor answered concerns and		**Motivation:** 70.1% required more information about treatment options or decisions, 61% sought reassurance that diagnosis or treatment already suggested was appropriate, 32.5% required more information about their cancer, and 31.2% were dissatisfied with the level of information or communication received so far<br>**Satisfaction:** 39.2% believed SO doctor listened

For peer review only

			0% believed they did not, 52.9% believed SO doctor gave them more confidence and 7.8% believed less, 47.1% believed SO doctor made them feel more confident and 3.9% believed less, 41.2% believed SO doctor was more friendly and 0% believed less, 49% believed they received more information from SO doctor and 2% believed less, and 51% believed they received new information from SO doctor<br>**Treatment:** 41.6% of patients intended to change treatment, with 28.6% continuing with		

For peer review only

			their second oncologist to do this and 13% staying with their first oncologist. 9.1% of patients intended to continue with their second oncologist but not change their treatment, giving 37.7% of patients who intended to change their oncologist		
Van Dalen, 2001[53]	New patients at an orthopaedic surgical outpatient clinic at a university hospital seeking an SO (group A) and their first opinion consultants (group B)	To identify the characteristics, motivating factors and first consultant experiences of patients who seek second orthopaedic surgical outpatient opinions	**Characteristics:** Those who felt their relationship with their FO consultant was poorer, those whose FO consultant practiced nearer to the SO centre and those that visited an FO consultant working in a larger group of consultants had a higher		**Motivation (group A):** 84% patient wanted more information about treatment possibilities, 67% patient wanted more information about the condition, 61% FO consultant had no solution to the problem, 60% results of treatment were disappointing, 51% patient dissatisfied with FO consultant, 43% FO consultant offered no treatment, 40% patient believed the diagnosis was incorrect, 39% patient had no confidence

For peer review only

			likelihood of seeking an SO on their own initiative		in the FO consultant, 37% FO consultant found no substantive diagnosis, 26% patient hoped to get a different diagnosis, 16% family/friends had had good experience with a certain consultant, 12% patient disapproved of the recommended treatment, 9% patient was concerned about the diagnosis, and 8% family/friends had had good experience with a certain treatment **Motivation (according to group B):** 33% suggested the results of the treatment had been disappointing, 28% suggested the patient wanted more information about the treatment, 16% suggested the patient wanted more information about the condition, 7% suggested the patient disapproved of the recommended treatment, 3% suggested the patient was concerned about the diagnosis, and 2% suggested there were communication problems

For peer review only

Wieske, 2011[54]	Patients referred to the neurological day-care clinic of an academic medical centre for an SO or TR	To investigate long-term patient satisfaction with a day-care admission for a neurological SO or TR			**Satisfaction:** Decrease in satisfaction shown on visual analogue scale (VAS) ranging from 0 'not at all' to 10 'completely' when comparing level of satisfaction with referring physician and 2 years after SO (5.4 vs 5.3; -0.1) and when comparing level of satisfaction directly after SO and 2 years after SO (-2.6)
Wijers, 2010 [55]	Patients referred to the neurological day-care clinic of an academic medical centre for an SO or TR	To explore the expectations of patients who seek a neurological SO or TR, and to assess patient satisfaction with a day-care admission for such a consultation	Mean age 47 years, 55.2% female, median duration of symptoms 2 years	**Diagnosis/Treatment:** 56% received a new diagnosis and/or treatment advice	**Motivation:** 59% expected a new diagnosis or treatment, 28% expected an explanation, and 6% expected confirmation of their diagnosis or treatment<br>**Satisfaction:** Overall satisfaction with SO 7.4 on VAS ranging from 0 'not at all' to 10 'completely' compared to 5.5 with FO. Higher scores for SO than FO with all aspects of satisfaction (own involvement in the conversation, physician's information giving, own involvement in decision-making, physicians' emotional support, and

For peer review only

					general satisfaction). Patients who received a new diagnosis/treatment were equally as satisfied with the consultation as patients who did not (7.5 vs 7.4)

For peer review only

Appendix 3: Risk of bias assessment

Study	1	2	3	4	5	6	7	8	9	10	11	12	13	14
Annandale, 1998 [24]	Y	Y	NR	Y	N	N	NA	NA	Y	NA	Y	N	NA	N
Benson, 2001 [25]	Y	Y	NR	Y	N	N	NA	NA	Y	NA	Y	N	NA	N
Cecon, 2019 [26]	Y	Y	Y	Y	N	N	NA	NA	Y	NA	Y	N	NA	N
Clauson, 2002 [27]	Y	Y	NR	Y	Y	N	NA	NA	Y	NA	Y	N	NA	N
Fuchs, 2017 [28]	Y	Y	N	Y	N	N	NA	NA	Y	NA	Y	N	NA	N
Gologorsky, 2013 [29]	Y	Y	Y	Y	N	N	NA	NA	Y	NA	Y	N	NA	N
Groß, 2017 [30]	Y	Y	Y	Y	Y	N	NA	NA	Y	NA	Y	N	NA	Y
Katz, 2017 [31]	Y	Y	Y	Y	N	N	NA	NA	Y	NA	Y	N	NA	Y
Kurian, 2017 [32]	Y	Y	Y	Y	Y	N	NA	NA	Y	NA	Y	N	NA	Y
Mellink, 2003 [33]	Y	Y	Y	Y	Y	N	NA	NA	Y	NA	Y	N	NA	N
Mellink, 2006 [34]	Y	Y	Y	Y	Y	Y	NA	NA	Y	NA	Y	N	Y	N
Meyer, 2015 [35]	Y	Y	Y	Y	Y	N	NA	NA	Y	NA	Y	N	NA	N
Mordechai, 2015 [36]	Y	Y	Y	Y	Y	N	NA	NA	Y	NA	Y	N	NA	N
Morrow, 2009 [37]	Y	Y	Y	Y	Y	N	NA	NA	Y	NA	Y	N	NA	N
Mustafa, 2002 [38]	Y	Y	Y	Y	N	N	NA	NA	Y	NA	Y	N	NA	N

Study	1	2	3	4	5	6	7	8	9	10	11	12	13	14
Okamoto, 2013 [39]	Y	Y	Y	Y	N	N	NA	NA	Y	NA	Y	N	NA	N
Philip, 2010 [40]	Y	Y	Y	Y	N	N	NA	NA	Y	NA	Y	N	NA	N
Radhakrishnan, 2017 [41]	Y	Y	Y	Y	Y	N	NA	NA	Y	NA	Y	N	NA	Y
Ramsey, 2011 [42]	Y	Y	Y	Y	Y	Y	NA	NA	Y	NA	Y	N	Y	Y
Sato, 1999 [43]	Y	Y	Y	Y	Y	N	NA	NA	Y	NA	Y	N	NA	Y
Schook, 2014 [44]	Y	Y	Y	Y	Y	N	NA	NA	Y	NA	Y	N	NA	N
Shmueli, 2016 [12]	Y	Y	Y	Y	Y	N	NA	NA	Y	NA	Y	N	NA	Y
Shmueli, 2017 [45]	Y	Y	Y	Y	Y	N	NA	NA	Y	NA	Y	N	NA	N
Shmueli, 2019 [46]	Y	Y	Y	Y	N	N	NA	NA	Y	NA	Y	N	NA	Y
Shmueli, 2019a [47]	Y	Y	Y	Y	Y	N	NA	NA	Y	NA	Y	N	NA	N
Sutherland. 1989 [48]	Y	Y	Y	Y	N	N	NA	NA	Y	NA	Y	N	NA	Y
Sutherland. 1994 [49]	Y	Y	Y	Y	N	N	NA	NA	Y	NA	Y	N	NA	Y
Tam, 2014 [51]	Y	Y	Y	Y	Y	N	NA	NA	Y	NA	Y	N	NA	N
Tam, 2005 [50]	Y	Y	Y	Y	Y	N	NA	NA	Y	NA	Y	N	NA	Y
Tattersall, 2009 [52]	Y	Y	Y	Y	N	N	NA	NA	Y	NA	Y	N	NA	N
Van Dalen, 2001 [53]	Y	Y	Y	Y	Y	N	NA	NA	Y	NA	Y	N	NA	Y

For peer review only - http://bmjopen.bmj.com/site/about/guidelines.xhtml

Study	1	2	3	4	5	6	7	8	9	10	11	12	13	14
Wieske, 2011 [54]	Y	Y	N	Y	Y	Y	NA	NA	Y	NA	Y	N	N	Y
Wijers, 2010 [55]	Y	Y	Y	Y	Y	N	NA	NA	Y	NA	Y	N	NA	Y

Y, Yes; N, No; NR, Not Reported; NA, Not Applicable

Study quality assessment

Study quality was assessed using the NIH Quality Assessment Tool for Observational Cohort and Cross-Sectional Studies.[34] The following 14 questions were answered for each study:

1. Was the research question or objective in this paper clearly stated?

2. Was the study population clearly specified and defined?

3. Was the participation rate of eligible persons at least 50%?

4. Were all the subjects selected or recruited from the same or similar populations (including the same time period)? Were inclusion and exclusion criteria for being in the

study prespecified and applied uniformly to all participants?

5. Was a sample size justification, power description, or variance and effect estimates provided?

6. For the analyses in this paper, were the exposure(s) of interest measured prior to the outcome(s) being measured?

7. Was the timeframe sufficient so that one could reasonably expect to see an association between exposure and outcome if it existed?

8. For exposures that can vary in amount or level, did the study examine different levels of the exposure as related to the outcome (e.g., categories of exposure, or exposure measured as continuous variable)?

For peer review only

9. Were the exposure measures (independent variables) clearly defined, valid, reliable, and implemented consistently across all study participants?

10. Was the exposure(s) assessed more than once over time?

11. Were the outcome measures (dependent variables) clearly defined, valid, reliable, and implemented consistently across all study participants?

12. Were the outcome assessors blinded to the exposure status of participants?

13. Was loss to follow-up after baseline 20% or less?

14. Were key potential confounding variables measured and adjusted statistically for their impact on the relationship between exposure(s) and outcome(s)?

For peer review only

PRISMA 2009 Checklist

Section/topic	#	Checklist item	Reported on page #
**TITLE**			
Title	1	Identify the report as a systematic review, meta-analysis, or both.	1
**ABSTRACT**			
Structured summary	2	Provide a structured summary including, as applicable: background; objectives; data sources; study eligibility criteria, participants, and interventions; study appraisal and synthesis methods; results; limitations; conclusions and implications of key findings; systematic review registration number.	2
**INTRODUCTION**			
Rationale	3	Describe the rationale for the review in the context of what is already known.	3
Objectives	4	Provide an explicit statement of questions being addressed with reference to participants, interventions, comparisons, outcomes, and study design (PICOS).	3
**METHODS**			
Protocol and registration	5	Indicate if a review protocol exists, if and where it can be accessed (e.g., Web address), and, if available, provide registration information including registration number.	N/A
Eligibility criteria	6	Specify study characteristics (e.g., PICOS, length of follow-up) and report characteristics (e.g., years considered, language, publication status) used as criteria for eligibility, giving rationale.	5
Information sources	7	Describe all information sources (e.g., databases with dates of coverage, contact with study authors to identify additional studies) in the search and date last searched.	5
Search	8	Present full electronic search strategy for at least one database, including any limits used, such that it could be repeated.	5
Study selection	9	State the process for selecting studies (i.e., screening, eligibility, included in systematic review, and, if applicable, included in the meta-analysis).	6
Data collection process	10	Describe method of data extraction from reports (e.g., piloted forms, independently, in duplicate) and any processes for obtaining and confirming data from investigators.	6
Data items	11	List and define all variables for which data were sought (e.g., PICOS, funding sources) and any assumptions and simplifications made.	6
Risk of bias in individual studies	12	Describe methods used for assessing risk of bias of individual studies (including specification of whether this was done at the study or outcome level), and how this information is to be used in any data synthesis.	6
Summary measures	13	State the principal summary measures (e.g., risk ratio, difference in means).	6
Synthesis of results	14	Describe the methods of handling data and combining results of studies, if done, including measures of consistency (e.g., I^2) for each meta-analysis.	6

For peer review only - http://bmjopen.bmj.com/site/about/guidelines.xhtml

PRISMA 2009 Checklist

Section/topic	#	Checklist item	Reported on page #
Risk of bias across studies	15	Specify any assessment of risk of bias that may affect the cumulative evidence (e.g., publication bias, selective reporting within studies).	6
Additional analyses	16	Describe methods of additional analyses (e.g., sensitivity or subgroup analyses, meta-regression), if done, indicating which were pre-specified.	6
**RESULTS**			
Study selection	17	Give numbers of studies screened, assessed for eligibility, and included in the review, with reasons for exclusions at each stage, ideally with a flow diagram.	6
Study characteristics	18	For each study, present characteristics for which data were extracted (e.g., study size, PICOS, follow-up period) and provide the citations.	6
Risk of bias within studies	19	Present data on risk of bias of each study and, if available, any outcome level assessment (see item 12).	7
Results of individual studies	20	For all outcomes considered (benefits or harms), present, for each study: (a) simple summary data for each intervention group (b) effect estimates and confidence intervals, ideally with a forest plot.	7-9
Synthesis of results	21	Present results of each meta-analysis done, including confidence intervals and measures of consistency.	7-9
Risk of bias across studies	22	Present results of any assessment of risk of bias across studies (see Item 15).	29
Additional analysis	23	Give results of additional analyses, if done (e.g., sensitivity or subgroup analyses, meta-regression [see Item 16]).	n/a
**DISCUSSION**			
Summary of evidence	24	Summarize the main findings including the strength of evidence for each main outcome; consider their relevance to key groups (e.g., healthcare providers, users, and policy makers).	9
Limitations	25	Discuss limitations at study and outcome level (e.g., risk of bias), and at review-level (e.g., incomplete retrieval of identified research, reporting bias).	10
Conclusions	26	Provide a general interpretation of the results in the context of other evidence, and implications for future research.	12
**FUNDING**			
Funding	27	Describe sources of funding for the systematic review and other support (e.g., supply of data); role of funders for the systematic review.	19

From: Moher D, Liberati A, Tetzlaff J, Altman DG, The PRISMA Group (2009). Preferred Reporting Items for Systematic Reviews and Meta-Analyses: The PRISMA Statement. PLoS Med 6(6): e1000097. doi:10.1371/journal.pmed1000097

For more information, visit: **www.prisma-statement.org**.

For peer review only - http://bmjopen.bmj.com/site/about/guidelines.xhtml
